# Stoichiometry-Dependent Structural Defects in Low-Generation PAMAM Dendrimers

**DOI:** 10.1021/acsomega.6c08154

**Published:** 2026-09-30

**Authors:** Chun T. Huang, Cesar L. Petzhold

**Affiliations:** Institute of Chemistry, 28124 Federal University of Rio Grande do Sul, Av. Bento Gonçalves, 9500, Porto Alegre, Rio Grande do Sul 90650-001, Brazil

## Abstract

Low-generation PAMAM dendrimers synthesized via a divergent approach from EDA cores were characterized to assess stoichiometry-dependent structural defects and product heterogeneity. MALDI-TOF MS, RP-HPLC-UV, SEC, and ^1^H NMR were employed as complementary techniques to assign and compare defect-related signatures, including trailing-generation species, intramolecular cyclization, and intermolecularly linked dendritic byproducts and/or higher-mass linked dendritic populations. Theoretical molecular weights were estimated using a modified Carothers equation and compared with experimental descriptors, although these comparisons were interpreted as descriptive stoichiometric trends rather than quantitative validation of the theoretical treatment. MALDI-TOF MS revealed repetitive fragment intervals (Δ*m/z* = 28, 60, 114), consistent with incomplete amidation, cyclization, and retro-Michael reactions. RP-HPLC-UV was used as an orthogonal semi-quantitative chromatographic fingerprinting technique to compare sample heterogeneity under identical analytical conditions. SEC provided apparent hydrodynamic-size information, and SEC-derived molecular-weight-related descriptors were interpreted cautiously due to the compact architecture of dendrimers and calibration with linear polymer standards, while ^1^H NMR gave semi-quantitative information on branched and bridged amide environments. The fraction of non-bridged species (*f*_0_) showed a trend consistent with theoretical expectations, although the limited number of stoichiometric conditions precludes rigorous statistical validation. These findings highlight the critical role of stoichiometric control in minimizing defects and reducing structural heterogeneity in low-generation PAMAM products, and emphasize the value of complementary analytical techniques for semi-quantitative assessment of dendrimer heterogeneity.

## Graphical abstract

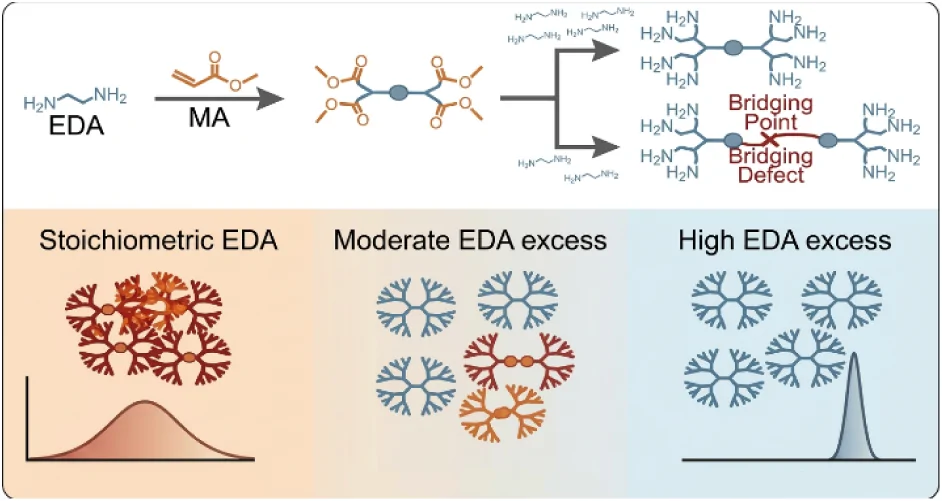

## 1 Introduction

Poly­(amidoamine) (PAMAM) dendrimers are a class of synthetic macromolecules characterized by their highly branched, tree-like architecture and nominally low dispersity. Originally developed by Tomalia and coworkers,^1–3^ PAMAM dendrimers have garnered significant attention for applications in drug delivery, catalysis, nanotechnology, and molecular diagnostics^4,5^ due to their well-defined structure and tunable surface functionalities. However, the structural ideality implied by a nominal dendrimer generation should be regarded as a synthetic target rather than an automatic outcome of divergent synthesis. Practical syntheses often produce mixtures containing structurally related species, including incompletely grown, cyclized, or intermolecularly linked products.^1,2^ Therefore, even commercially available or low-generation PAMAM materials may contain structurally related impurities and defect populations, indicating that nominal dendrimer generation alone does not guarantee molecular uniformity.^6^

While higher-generation dendrimers (G≥3) have been extensively studied for their size-dependent properties,^1–3,7–9^ low-generation analogs (G-0.5 to G0) offer unique advantages in terms of synthetic accessibility, solubility, and functional versatility.^10–12^ However, even at early stages of dendrimer growth, structural imperfections such as incomplete amidation, incomplete branching, trailing generations, intramolecular cyclization, and intermolecular bridging can compromise molecular uniformity and functional performance. These defects may generate a distribution of PAMAM-related products rather than a single discrete dendrimer structure.^1,13,14^ Similar heterogeneity has been reported for PAMAM materials using chromatographic and mass-spectrometric methods, including species assigned to trailing generations, dimers, oligomeric byproducts, and other defect-containing populations.^6,15^ Accordingly, low-generation PAMAM products represent a useful model system for evaluating how reagent stoichiometry affects defect formation during divergent dendrimer synthesis.

Studies have demonstrated that matrix-assisted laser desorption ionization time-of-flight mass spectrometry (MALDI-TOF MS) is particularly effective in identifying structural defects in PAMAM dendrimers, including fragmentation products and covalently linked aggregates.^5,13^ Peterson et al. provided a detailed MALDI-TOF MS analysis of PAMAM dendrimers up to generation 5, revealing mass deviations and fragmentation patterns associated with cyclization and retro-Michael reactions.^13^ Kallos et al. employed electrospray ionization (ESI-MS) to determine molecular weights and dispersity indices, achieving high accuracy and demonstrating the utility of deconvolution algorithms for complex dendritic mixtures.^14^ Ottaviani et al. further explored dendrimer morphology using electron paramagnetic resonance, correlating Cu­(II) complexation with dendrimer generation and internal accessibility.^7^ In parallel, high-performance liquid chromatography (HPLC) based methods have been used as complementary quality-control tools for PAMAM and PAMAM-derived materials, providing chromatographic fingerprints sensitive to generation, surface functionality, and structural heterogeneity.^15,16^

A distinction must be made between ideal dendrimers, deliberately constructed megamers, and linked dendritic byproducts that may arise during divergent synthesis. Ideal PAMAM dendrimers are commonly described as discrete core–shell macromolecules composed of a defined core, generational branch-cell shells, and terminal functional groups, with molecular weights and surface-group numbers that can be predicted as a function of generation.^17–19^ By contrast, bridged species are operationally defined as covalently connected products formed by intermolecular coupling between dendrimeric or partially dendrimeric units. Such linked products may include dimers, small oligomeric dendritic byproducts, or broader dendritic populations, consistent with previous descriptions of dendrimer multiples and interdendrimer surface reactions.^17,19^

Dendrimer-derived megamers have been described as higher-complexity architectures constructed from dendritic building blocks, including statistical megameric clusters and more structure-controlled core–shell tecto­(dendrimers). These architectures may be produced through direct covalent linking, electrostatically directed self-assembly followed by covalent fixation, or other supramolecular assembly approaches.^18,19^ Accordingly, the higher-mass or strongly retained populations detected in the present work are not automatically assigned as well-defined megamers. They may instead correspond to statistically formed linked dendritic species with heterogeneous composition, connectivity, and molar mass. Therefore, the term “megamer-like species” when used, is used conservatively in this study and only for higher-mass or strongly retained populations consistent with linked dendritic structures. The broader focus of this work is the formation of stoichiometry-dependent structural defects rather than the deliberate synthesis or definitive identification of well-defined megamer architectures.

Advances in high-resolution mass spectrometry have further improved the detection of dendrimer fragmentation and aggregation, enabling precise molecular weight profiling even in complex mixtures. MALDI-TOF MS and ESI-MS remain key techniques for identifying incomplete branching, intramolecular cyclization, and retro-Michael rearrangements in low-generation PAMAM dendrimers.^13,14,20–22^ Analytical challenges linked to matrix effects and ionization biases have been mitigated through optimized sample preparation, ionization conditions, and fragmentation protocols.^13,14,20–23^ Furthermore, aggregation behavior and surface interactions significantly influence molecular uniformity and performance, reinforcing the need for rigorous purification and characterization strategies.^13,14,20^ However, MALDI-TOF MS may underrepresent poorly ionizing or high-mass bridged species, whereas ESI-MS can be affected by charge-state complexity and deconvolution assumptions. Therefore, mass spectrometry alone may not provide a fully quantitative description of low-generation PAMAM mixtures.

SEC, although widely used for polymer analysis, presents limitations in accurately determining the molecular weights of dendrimers due to their compact, globular architecture and deviation from linear calibration standards. Multi-detector SEC and universal calibration approaches improve accuracy, but still depend on the choice of standards and solvent conditions.^1,8,24,25^ In low-generation PAMAM systems, SEC is therefore more useful for comparing apparent hydrodynamic-size distributions and detecting higher-apparent-size populations than for assigning absolute molecular weights. HPLC can provide an additional fingerprint of sample heterogeneity and purification efficiency, although retention depends on interactions with the stationary phase and does not directly translate into molecular weight.^6,15–16^ ^1^H and ^13^C NMR spectroscopy remain valuable tools for estimating end-group conversion, branching efficiency, residual monomer or reagent content, and internal dynamics, especially when combined with multidimensional techniques and relaxation analysis.^23,25–27^

The Carothers equation, originally developed for linear step-growth polymerizations, has been successfully adapted to dendritic systems to estimate apparent molecular-weight trends and contextualize defect formation.^28^ In divergent PAMAM synthesis, the use of excess ethylenediamine (EDA) is a known strategy to favor amidation, suppress intermolecular bridging, and improve terminal-group conversion. However, the extent to which EDA excess improves structural quality must be balanced against purification demands and residual-reagent removal. Advances in dendrimer chemistry have enabled precise control over branching, defect suppression, and functional group placement, facilitating the design of dendrimer–drug conjugates, polymer–dendrimer hybrids, and dendritic networks.^29^ These architectures have demonstrated versatility in catalysis, biosensing, and nanomedicine, with dendrimer–metal complexes showing particular promise.^29^ High-resolution mass spectrometry and surface interaction studies have further illuminated dendrimer–environment dynamics, enhancing structural characterization and synthetic optimization.^29,30^ Recent studies have also localized critical molar ratio intervals for highly branched Michael addition networks, supporting the use of Carothers-based approaches to estimate gelation boundaries and discuss transitions between dendritic and highly linked architectures.^31^ Thus, stoichiometry is not only a synthetic parameter but also a practical variable that can be correlated with analytical signatures of incomplete growth, bridging, and purification efficiency.

In this study, we investigate low-generation PAMAM dendrimer-related products obtained under different EDA stoichiometric conditions. Using MALDI-TOF MS, SEC, RP-HPLC-UV, and ^1^H NMR, we compare complementary analytical responses, evaluate defect-related signatures, and assess the impact of EDA excess on branching-related environments and apparent structural homogeneity. Theoretical molecular weights were estimated using an adapted Carothers equation,^32,33^ and the fraction of non-bridged species was estimated from a statistical model and compared with semi-quantitative ^1^H NMR descriptors.

The novelty of this work does not lie in the general use of excess EDA, which is already recognized in PAMAM synthesis, but in the integrated comparison of mass-spectrometric, chromatographic, hydrodynamic, and NMR descriptors to evaluate how EDA stoichiometry modulates defect-related signatures in low-generation PAMAM products.

## 2 Experimental Section

### 2.1 Materials

Methyl acrylate (MA, 99%) and ethylenediamine (EDA, 99%) were purchased from Sigma-Aldrich and Neon, respectively, and used without further purification. Methanol (analytical grade), and deuterium oxide (D_2_O) were used as solvents for synthesis and NMR analysis. An independent PAMAM benchmark sample was provided by Dorf Ketal Brasil Ltda. and used only as an internal chromatographic comparison sample in RP-HPLC-UV analyses. This material was not used as a certified analytical standard, calibration material, or absolute purity reference.

### 2.2 Synthesis of Amine-Terminated PAMAM Dendrimer-Related Products and Linked Dendritic Species

Amine-terminated PAMAM structures, encompassing generation 0 dendrimer and linked dendritic analogues, were synthesized via a two-step divergent sequence involving Michael addition of MA to EDA, followed by amidation. First, the ester-terminated intermediate was prepared via Michael addition of MA to EDA. Briefly, 534.6 mmol of MA was added dropwise over 1 h to a methanolic solution of EDA (1.0 mmol mL^–1^), containing 121.6 mmol of EDA, at 40 °C under magnetic stirring. After the addition of MA, the reaction mixture was maintained at 40 °C for 24 h. The consumption of primary amine groups was qualitatively evaluated by the Rimini test, which showed a negative response at the end of the reaction. Excess MA and volatile components were removed under reduced pressure, affording the ester-terminated intermediate with a final yield of 92.3%.

The obtained ester-terminated intermediate was then used in the amidation step to prepare D0, D1, and D2. For each synthesis, approximately 26.3 mmol of the ester-terminated intermediate, corresponding to approximately 105.2 mmol of ester terminal groups, was heated to 50 °C under magnetic stirring. A methanolic solution of EDA was then added dropwise over 1 h. To evaluate the influence of the EDA molar ratio on branching efficiency and the formation of covalently bridged or oligomeric dendritic byproducts, three conditions were employed: a stoichiometric ratio (sample D0) and two excess conditions corresponding to 150% (D1) and 650% (D2) molar excess of EDA relative to the ester terminal groups. For D0, 104.9 mmol of EDA was added as a 1.0 mmol mL^–1^ methanolic solution, corresponding to approximately 1.0 equiv of EDA per ester terminal group. For D1, 263 mmol of EDA was added as a 2.83 mmol mL^–1^ methanolic solution, corresponding to approximately 2.5 equiv of EDA per ester terminal group. For D2, 789 mmol of EDA was added as a 15 mmol mL^–1^ methanolic solution, corresponding to approximately 7.5 equiv of EDA per ester terminal group. After EDA addition, each reaction mixture was maintained at 50 °C for 24 h under magnetic stirring. Volatile components were then partially removed under reduced pressure at temperatures not exceeding 50 °C.

In this work, the terms stoichiometric ratio or 0%, 150%, and 650% EDA excess refer to the molar amount of EDA molecules relative to ester terminal groups, rather than to the total number of primary amine functional group equivalents. No additional purification was performed, and the resulting materials were characterized as obtained. Residual EDA and methanol were still detectable by ^1^H NMR, indicating that the reduced-pressure treatment was insufficient for complete removal of low-molecular-weight volatile components. No *in situ* reaction monitoring was performed during the amidation step; therefore, the reaction time was fixed at 24 h for all samples to enable comparison under identical conditions. The final materials were analyzed by ^1^H NMR after reduced-pressure treatment. Reliable isolated yields were not determined for D0, D1, and D2 because residual EDA and methanol remained detectable in the as-obtained materials, preventing accurate gravimetric determination of product mass. The resulting products were characterized to assess structural variations and the extent of formation of linked dendritic species.

### 2.3 Size Exclusion Chromatography (SEC)

SEC analyses were performed using a Viscotek VE2001 system equipped with a refractive index detector (RI VE-3580), a Shodex SB-807G guard column, four Shodex analytical columns arranged as 2 × SB-807 HQ and 2 × SB-806M HQ. The system was calibrated using monodisperse poly­(ethylene oxide) standards. During the chromatographic run, 0.1 M aqueous NaNO_3_ was used as the mobile phase at a flow rate of 0.5 mL min^–1^ and a temperature of 35 °C. Samples were prepared by dissolving 20 mg of material in 4 mL of 0.1 M aqueous NaNO_3_, corresponding to a nominal concentration of 5 mg mL^–1^. Before injection, the solutions were filtered through a Chromafil Xtra PVDF-45/25 membrane filter with a pore size of 0.45 μm. The injection volume was 100 μL, corresponding to an approximate column loading of 500 μg per injection. All samples were prepared at the same nominal concentration and injected under identical chromatographic conditions.

### 2.4 Reversed-Phase High-Performance Liquid Chromatography with UV Detection (RP-HPLC-UV)

RP-HPLC-UV analyses were performed using a Dionex Ultimate 3000 liquid chromatograph coupled to a UV-Vis detector. Separations were carried out on a Dionex Acclaim 120 C8 column, 250 mm × 4.6 mm, 5 μm. Samples were prepared at 1000 ppm in the initial mobile-phase composition, 95:5 water/acetonitrile, filtered through a 0.22 μm PVDF membrane filter, and injected into the chromatographic system. For each sample, RP-HPLC-UV analyses were performed in triplicate under identical chromatographic conditions to assess the reproducibility of the chromatographic fingerprint, retention-window distribution, and relative UV area contributions. Representative chromatograms are shown in the figures, whereas the relative UV area contributions are reported as mean values with the corresponding standard deviations.

Mobile phase A consisted of ultrapure water, Merck Milli-Q Integral 5, and mobile phase B consisted of acetonitrile. The column temperature was maintained at 30 °C, the injection volume was 10 μL, and the mobile-phase flow rate was 1.0 mL/min. The gradient program was as follows: 5% B at 0 min, increased to 80% B over 30 min, increased to 95% B over 6 min, and returned to 5% B over 10 min. UV detection was performed at 210 nm.

The independent PAMAM benchmark sample was prepared and analyzed using the same sample concentration, filtration procedure, injection volume, column, mobile phase, gradient, flow rate, temperature, and UV detection wavelength used for D0, D1, and D2. The comparison with this benchmark was restricted to chromatographic retention behavior and relative UV area distribution under identical analytical conditions.

RP-HPLC-UV was used as a complementary fingerprinting technique to evaluate sample heterogeneity, retention-profile changes, and chromatographic homogeneity. Because retention in reversed-phase chromatography depends on analyte–stationary-phase interactions and not solely on molecular weight, RP-HPLC-UV data were interpreted comparatively and semi-quantitatively rather than as absolute molecular-weight distributions. The reproducibility assessment was restricted to the chromatographic response under identical analytical conditions and was not used to assign absolute molar fractions or absolute species abundances.

### 2.5 Matrix-Assisted Laser Desorption Ionization Time-of-Flight Mass Spectrometry (MALDI-TOF MS)

MALDI-TOF MS was conducted using an Autoflex Speed TOF MS instrument (Bruker) in positive linear mode. Samples (10–20 mg) were dissolved to 4 mg/mL and mixed with 2,5-dihydroxybenzoic acid (10 mg/mL in 50% methanol with 0.1% trifluoroacetic acid). Spectra were acquired over an *m/z* range of 498–3428.

Peaks corresponding to the target low-generation PAMAM dendrimer and higher-mass defect-related populations were assigned when consistent with expected mass ranges. Relative peak intensities were used only as semi-quantitative descriptors of ion contribution within each spectrum, not as absolute molar abundances.

Independent sample preparations were not used to statistically assess the reproducibility of MALDI-TOF MS intensity distributions. Therefore, relative ion contributions are reported only as intra-spectrum semi-quantitative descriptors obtained under identical sample-preparation and acquisition conditions, and not as statistically validated abundance values. Accordingly, MALDI-TOF MS was used primarily for mass-pattern assignment and qualitative/semi-quantitative comparison of assigned ion contributions within each spectrum, whereas chromatographic reproducibility was assessed independently by replicate RP-HPLC-UV analyses.

### 2.6 Nuclear Magnetic Resonance (^1^H NMR)

Samples for nuclear magnetic resonance (^1^H NMR) spectroscopy were prepared by dissolving approximately 10–20 mg of each PAMAM-derived sample in 0.75 mL of deuterium oxide solvent.

^1^H NMR spectra were acquired using a Bruker spectrometer operating at 400 MHz, with data collected over a chemical shift range of −3 to 16 ppm. Spectral data were processed using standard procedures, including baseline correction and peak integration, to identify and assign signals corresponding to PAMAM dendritic structures, residual reagents, terminal groups, and defect-related environments.

NMR integration was used to estimate end-group conversion and branching-related features. However, because signals from ideal dendrimers, incompletely branched structures, and covalently bridged species may overlap, NMR-derived values were interpreted as semi-quantitative descriptors of structural quality rather than as absolute defect concentrations.

### 2.7 Molecular Weight Estimation

Theoretical molecular weights were estimated using the Carothers-type equation^32,33^ adapted for dendritic systems and linked dendritic species. Deviations from ideal values were attributed to incomplete branching, stoichiometric imbalance, intramolecular cyclization, and the possible formation of covalently bridged or oligomeric dendritic byproducts. Theoretical number-average (*M*_*n*_) and weight-average (*M*_*w*_) molecular weights were estimated using the Carothers-type equation^32,33^ adapted for polymerization of dendritic architectures:

$$M_{n}=M_{o}/\left(1-p\right)+M_{t}$$

$$M_{w}=M_{o}\left(1+p\right)/\left(1-p\right)+M_{t}$$

where *M*_0_ is the molecular weight of the structural unit, shown in the chemical structure, corresponding to the dendritic repeat unit used to represent the *n* repeating dendritic species, and was taken here as 460.30 g mol^–1^. The term *M*_*t*_ represents the contribution of the terminal groups, defined as the molecular weight of one methanol molecule. The parameter *p* is an operational stoichiometric imbalance parameter defined by the limiting functional-group ratio under each EDA condition. Specifically, *p* was calculated from the fraction of initially present reactive functional groups that are consumed when the limiting functional group is assumed to react completely. The parameter *p* decreases as the initial excess of EDA increases because it is normalized by the total initial number of reactive functional groups. Therefore, this parameter reflects the initial stoichiometric imbalance of the system and not the experimental extent of amidation.


$$p=\left(n_{\mathrm{ester},\mathrm{reacted}}+n_{\mathrm{amine},\mathrm{reacted}}\right)/\left(n_{\mathrm{ester},\mathrm{initial}}+n_{\mathrm{amine},\mathrm{initial}}\right)$$

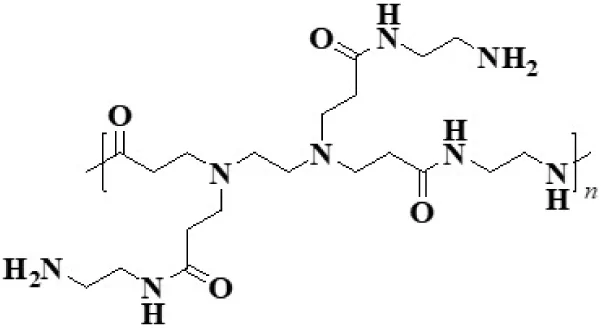
Since one amine group reacts with one ester group, and the ester groups are limiting:

$$p=\left(2n_{\mathrm{ester},\mathrm{reacted}}\right)/\left(n_{\mathrm{ester}}+n_{\mathrm{amine}}\right)$$

where *n*_ester_ = 4 for 1 mol of ester-terminated dendrimer and *n*_amine_ = 2 *n*_EDA_. Therefore:

$$p=8/\left(4+2n_{EDA}\right)$$

As a worked example, for sample D0, *n*_EDA_ = 4. Therefore, the total number of initial amine functional groups is:

$$n_{\mathrm{amine}}=2n_{EDA}=2\left(4\right)=8$$

Because the ester groups are limiting, four ester groups and four amine groups are assumed to react completely. Thus:

$$p=8/\left(4+2n_{EDA}\right)=8/\left(4+8\right)=0.667$$

The same calculation gives *p* = 0.333 for D1 and *p* = 0.125 for D2. The complete derivation of *p* for all samples is provided in Table S1.

These equations follow the classical Carothers approach for step-growth polymerization,^32,33^ modified to account for dendritic architecture, stoichiometric imbalance, and residual mass contributions. However, because *p* is derived from the initial functional-group balance rather than from experimentally measured conversion, the resulting theoretical molecular weights should be interpreted as descriptive Carothers-type estimates and not as experimentally determined molecular-weight values.

Experimental molecular-weight-related descriptors were obtained using SEC, MALDI-TOF MS, and ^1^H NMR. These techniques provided complementary insights into the molecular weight distribution, structural integrity, branching efficiency, and relative heterogeneity of the synthesized PAMAM-derived products.

SEC was used to compare hydrodynamic-size distributions, MALDI-TOF MS to assign mass-resolved species within the accessible *m/z* range, ^1^H NMR to evaluate conversion and branching-related signals, and RP-HPLC-UV to compare chromatographic fingerprints and chromatographic homogeneity. The results obtained by each method are presented and discussed in detail in the Results and Discussion section, where they are compared with the theoretical values derived from the adapted Carothers equations.

The parameter *n* was used as an apparent series index to describe groups of MALDI-TOF signals separated by mass intervals compatible with nominal PAMAM-related structural contributions. This notation was adopted for comparative purposes only and does not imply a definitive assignment of discrete oligomeric, dimeric, trimeric, or ideal dendritic structures.

### 2.8 Estimation of the Non-Bridged Species Fraction

The fraction of non-bridged species (*f*_0_) was estimated both experimentally and theoretically. Theoretical values were estimated using a statistical model adapted from Tomalia *et al*.,^1^ defined as:

$$fo={\left(1-P\right)}^{{N_{c}N_{r}}^{\left(G-1\right)}}$$

where *P* = 1/4*X*–1 is the probability of bridge formation, and *X* is the molar ratio of EDA to ester terminal groups. *N*_*c*_ is the core functionality (e.g., 4 for EDA), *N*_*r*_ is the number of reactive sites per branching unit (typically 2), and *G* is the dendrimer generation.

This exponential–exponential relationship highlights the strong dependence of *f*_0_ on generation and reagent stoichiometry. Large EDA excesses favor the formation of non-bridged dendritic structures, whereas lower excesses promote intermolecular bridging and increased structural heterogeneity, which may lead to covalently bridged species, oligomeric dendritic byproducts, or higher-mass linked dendritic populations.

The terms “bridged” and “unbridged” refer to the covalent connectivity between dendrimers. Species that originate from covalent cross-linking reactions, such as amidation between terminal groups, are considered bridged even if they later fragment into smaller units (e.g., dimers or trimers). In contrast, dendrimers that grow independently without forming covalent bonds with other dendrimers are classified as unbridged. This distinction is critical for interpreting both theoretical predictions and experimental data, especially when assessing branching efficiency and structural uniformity.

Experimental values of *f*_0_ were obtained by ^1^H NMR spectroscopy, based on the integration of aliphatic methylene proton signals corresponding to amide groups. Specifically, the ratio between the signal area of methylene protons associated with branched (non-bridged) amide groups and the total area of methylene protons from all amide groups (including both branched and bridged species) was used to estimate the apparent fraction of non-bridged dendritic environments. This approach provides a semi-quantitative measure of branching efficiency and was compared with theoretical estimates obtained from the statistical expression.

## 3 Results and Discussion

### 3.1 Stoichiometry-Dependent Defect Signatures and Structural Heterogeneity

The stoichiometry-dependent structural heterogeneity and analytical response of PAMAM dendrimers and defect-related dendritic species was investigated using three different EDA ratios. The experimental data, obtained from step-growth polymerization, are presented through SEC, ^1^H NMR, RP-HPLC-UV, and MALDI-TOF MS analyses.

In response to the limitations associated with assigning all non-ideal products as megameric structures, this discussion adopts a more conservative terminology. The term “dendrimer” is used for the target low-generation PAMAM structure, whereas “linked dendritic species”, “covalently bridged species”, “oligomeric dendritic byproducts”, and “megamer-like species” are used only when supported by the analytical evidence. Therefore, the present work is positioned as a comparative assessment of defect formation during low-generation PAMAM synthesis rather than as a definitive study of giant megamer architectures.

MALDI-TOF MS analysis revealed signals assigned to ideal dendrimer-related species together with higher-mass defect-related populations in samples synthesized with stoichiometric or moderately excess EDA. Peaks corresponding to species with higher *m/z* values than the target monomeric dendrimer were observed at *m/z* values significantly higher than the theoretical monomer mass, suggesting analytical signatures consistent with covalently bridged or oligomeric dendritic byproducts. These species are attributed to side reactions such as intermolecular amidation, incomplete amidation, and/or related coupling events that deviate from ideal dendrimer growth, as shown in Figure 1.

**Figure 1:**
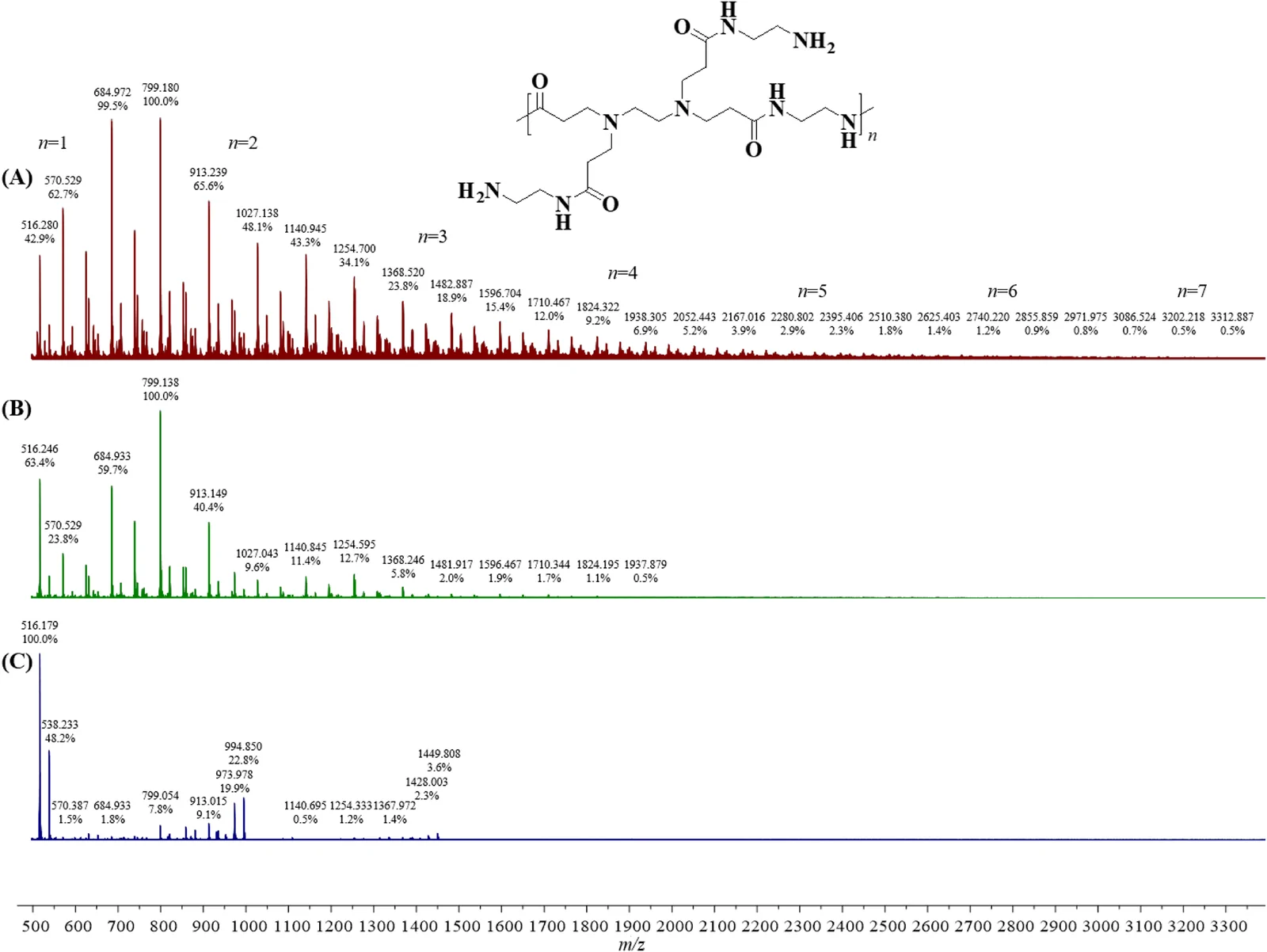
MALDI-TOF spectra of PAMAM dendrimers and defect-related dendritic species synthesized with different EDA excess: (A) D0, (B) D1, (C) D2. The main peaks are labeled with their experimentally observed *m/z* values and relative intensities. The inset shows a nominal PAMAM-related repeating structural motif used only as a guide for tentative mass-pattern assignments. The label *n* represents an apparent PAMAM-related mass-series index and should not be interpreted as dendrimer generation.

In the mass spectrum of D0, the molecular ion peak at *m/z* 516.280, 42.9% relative intensity, along with its protonated ([M + H]^+^) and sodium-adducted ([M + Na]^+^) forms, represented only 3.2% of the total MALDI ion contribution within the *m/z* range defined by peaks with at least 5% relative intensity. This indicates a low relative ion contribution from the ideal dendrimer-related signals under the MALDI-TOF MS conditions used. The prominent MALDI signals at *m/z* 684.972 and 799.180 were tentatively assigned to dimer-derived PAMAM defect species rather than to independent monomeric products, in agreement with previous reports describing structural defects and dimer/bridged species in PAMAM dendrimers. Considering the main D0 PAMAM-related ion at *m/z* 516.280, twice this mass minus 60 Da would give a covalently bridged dimer-related species near *m/z* 973. Within this mass-pattern interpretation, the ion at *m/z* 799.180 is consistent with a bridged dimer bearing one additional 60 Da defect, associated with intramolecular cyclization, EDA loss, or EDA-related covalent bridging, together with one 114 Da missing-arm/repeat-unit defect associated with incomplete Michael addition and/or retro-Michael-type processes. Similarly, the ion at *m/z* 684.972 is consistent with an analogous dimer-derived defect containing one additional 60 Da defect and two 114 Da missing-arm/repeat-unit defects. The 114 Da mass intervals have been associated with incomplete Michael addition and/or retro-Michael-type processes in PAMAM dendrimers, whereas 60 Da differences have been related to cyclization, EDA loss, or EDA-associated defects.^13,14,20–22^ Therefore, these assignments are proposed as representative tentative structures based on mass-pattern consistency, rather than as unique structural elucidations, considering the intrinsic limitations of MALDI-TOF MS for unambiguous structural assignment of isomeric or defect-containing dendrimer species. These repetitive mass intervals suggest extensive molecular imperfections, as shown in Figure 1A. The tentative MALDI-TOF MS assignments for the main ions observed in D0, D1, and D2 are summarized in Table S2 of the Supporting Information. The MALDI-TOF MS spectra used to estimate the apparent dendrimer-related ion contribution are provided in Figures S1–S3.

In contrast, D1 showed an increased relative ion contribution of ideal dendrimer-related signals to 10.5%, with a lower apparent contribution from higher-mass defect-related MALDI signals. The spectrum still exhibited moderate fragmentation, indicating partial suppression of defect formation, as shown in Figure 1B.

The sample D2 spectrum showed a higher apparent contribution from dendrimer-related MALDI signals, with 41.9% of the total ion signal assigned to ideal dendrimer-related MALDI signals. The dominant peak was assigned to the ideal dendrimer-related ion at *m/z* 516.179, with low relative ion contribution from higher-mass defect-related signals. Fragmentation was minimal, and defect-related MALDI signal contributions were substantially lower, as shown in Figure 1C.

Overall, the relative MALDI ion contribution of higher-mass defect-related signals decreased with increasing EDA excess. Thus, the sequence D0, D1, and D2 shows a progressive increase in dendrimer-related signal contribution and a concomitant decrease in apparent defect-related MALDI responses. Therefore, the MALDI-TOF MS data support a qualitative and semi-quantitative trend in which increasing EDA excess reduces the apparent relative ion contribution of defect-related signals.

Because MALDI-TOF MS ion intensities depend on matrix crystallization, desorption/ionization efficiency, adduct formation, fragmentation, and mass-dependent detection bias, these values were not treated as absolute molar fractions. Instead, they were used as semi-quantitative descriptors of the relative contribution of assigned signals within each spectrum.

In the expanded region of the MALDI-TOF mass spectrum, repetitive fragment intervals (Δ*m/z* = 28, 60, and 114) were observed, indicative of structural imperfections within the polymer, as shown in Figure 2. These mass differences are consistent with specific side reactions occurring during polymerization, as shown in Scheme 1: Δ*m/z* = 28 suggests incomplete amidation; Δ*m/z* = 60 is attributed to intramolecular cyclization or bridge formation; and Δ*m/z* = 114 corresponds to retro-Michael reactions or incomplete Michael addition.

**Figure 2:**
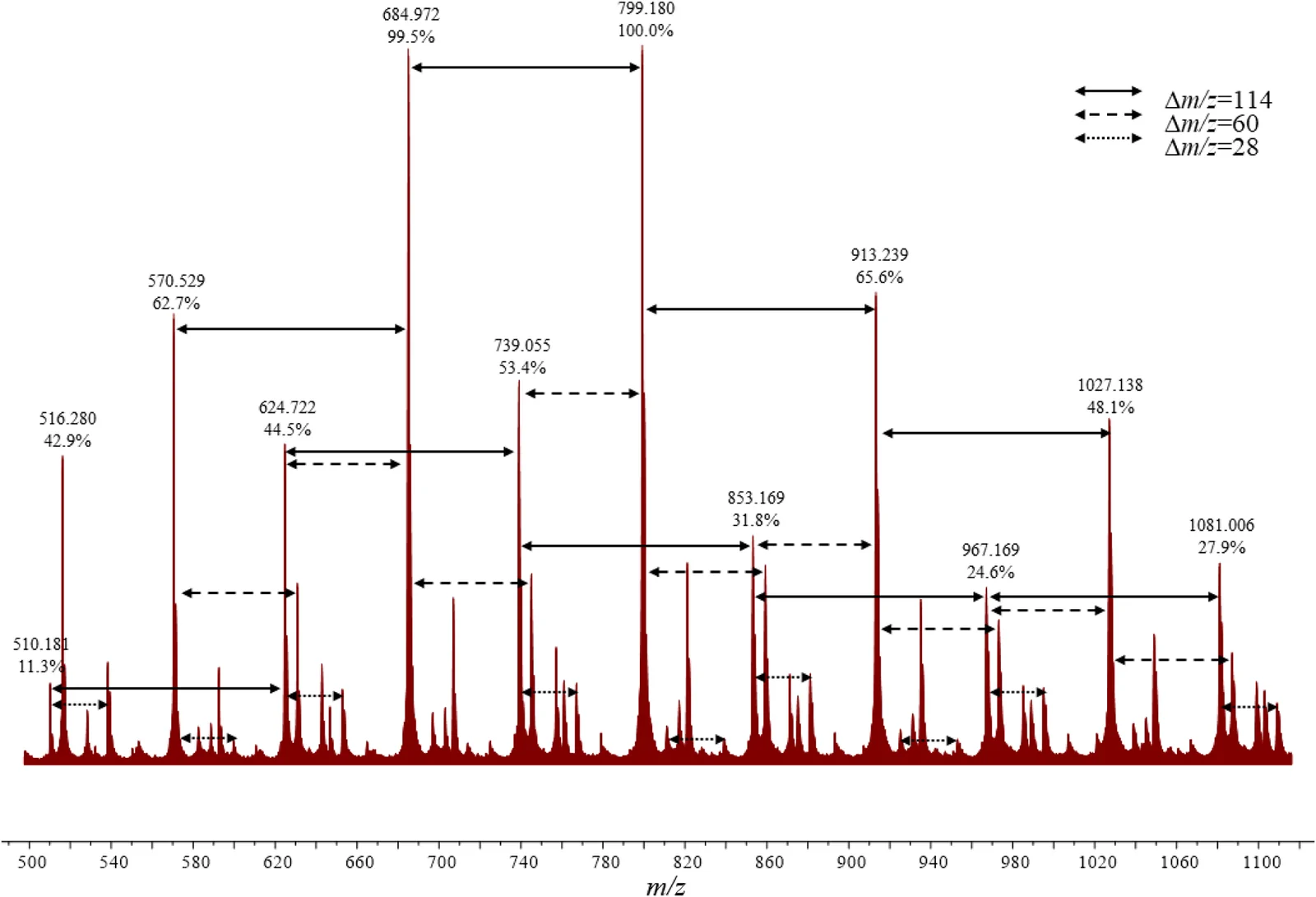
Expanded MALDI-TOF mass spectrum of the sample D0 (498–1116 *m/z*), showing repetitive fragment intervals (Δ*m/z* = 28, 60, and 114) associated with structural imperfections.

**Scheme 1:**
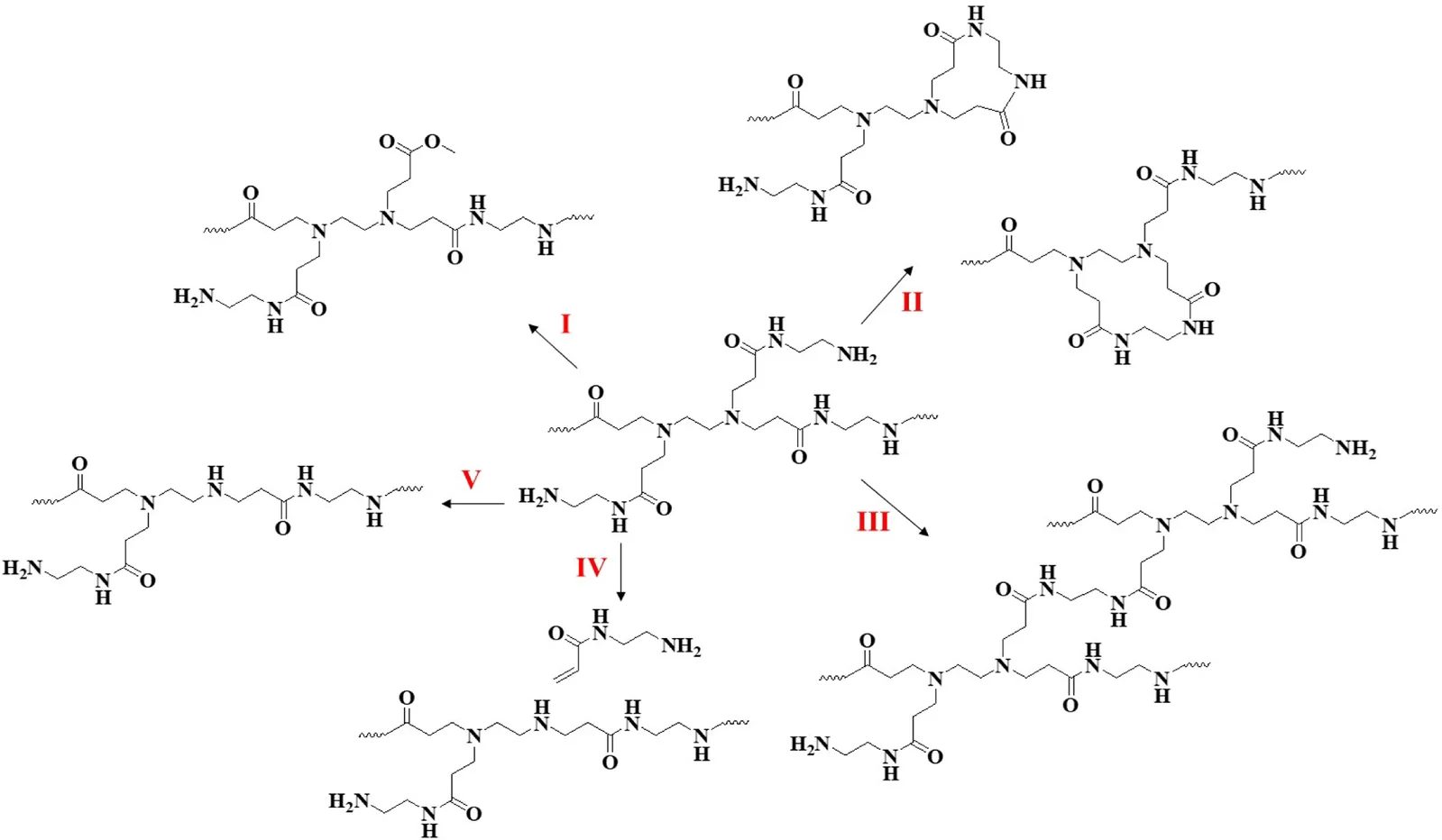
Main Side Reactions Leading to Structural Defects in PAMAM Dendrimers and Linked Dendritic Species^a^

a (I) incomplete amidation, (II) intramolecular cyclization, (III) bridge formation, (IV) retro-Michael reaction, and (V) incomplete Michael addition.

The presence of these recurring fragments supports the occurrence of side reactions that deviate from the ideal step-growth mechanism, contributing to the observed molecular heterogeneity. Such defects are particularly relevant in dendrimer synthesis because steric hindrance, local concentration effects, and reduced accessibility of terminal groups may alter the relative rates of Michael addition and amidation. This framework supports the hypothesis that dendrimer growth is not strictly stepwise at the molecular level, and that autoreactive intermediates may propagate structural heterogeneity in both dendrimers and linked dendritic byproducts.

A linear correlation (R^2^ = 0.9982) was observed between the molar excess of EDA and the relative MALDI-TOF MS ion contribution of ideal dendrimer-related signals. Given that the apparent contribution of defect-related MALDI signals tends to decrease as the ideal dendrimer-related ion contribution increases, the same trend is consistent with a lower contribution from signals assigned to covalently bridged species, oligomeric dendritic byproducts, and other defect-related populations.

These results reinforce the role of stoichiometric control in directing the outcome of dendritic synthesis, where low EDA excess is associated with stronger analytical signatures of bridging and structural heterogeneity, while high excess is associated with increased dendrimer-related signal contribution and reduced defect-related responses. Although the dataset is limited to three synthesis conditions, the consistency of the correlations supports the hypothesis that EDA excess can serve as a useful synthetic parameter for tuning the structural profile of PAMAM products. Because the regression is based on only three experimental points, it should be interpreted as a preliminary correlation rather than as a validated predictive model. Additional syntheses using intermediate EDA ratios would be required to establish a statistically robust model.

RP-HPLC-UV was introduced as a complementary technique to evaluate chromatographic fingerprints, sample heterogeneity, and the practical effect of EDA excess on the relative chromatographic response of PAMAM-related components. Because the separation was performed on a C8 reversed-phase column with UV detection at 210 nm, retention is expected to depend on differences in polarity, hydrophobic interaction with the stationary phase, molecular connectivity, and possibly aggregation behavior. Therefore, HPLC peak areas were interpreted comparatively and semi-quantitatively, not as absolute molar fractions.

The chromatographic profiles showed four main retention windows: low-retention species at 9.5–10.0 min, the main PAMAM-related retention window at 10.0–10.5 min, intermediate defect-related species at 10.5–12.8 min, and late-eluting strongly retained species at 14.0–14.5 min, possibly including oligomeric, bridged, or aggregate-like PAMAM-related populations. The relative UV area contributions were calculated by normalizing the integrated UV area of each defined retention window to the total integrated UV area within the selected 9.5–15.0 min chromatographic interval. The analyses were performed in triplicate, and the relative UV area contributions are reported as mean ± standard deviation in Table 1. Representative chromatograms are shown in Figure 3. Based on reversed-phase HPLC behavior reported for PAMAM-related systems, lower-retention defect populations, including trailing-generation species, are commonly observed before or near the main PAMAM-related peak/window, whereas dimeric or more strongly retained dendritic byproducts may appear at longer retention times. However, these assignments should be regarded as operational retention-window assignments and not as definitive structural identifications unless supported by isolation and orthogonal molecular-weight or mass analysis.^6,15,16^

**Table 1:** Relative UV Area Contributions in Defined Retention Windows for PAMAM Samples Synthesized with Different EDA Excess^a^

Sample Code	Relative UV area 9.5–10.0 min (%)	Relative UV area 10.0–10.5 min (%)	Relative UV area 10.5–12.8 min (%)	Relative UV area 14.0–14.5 min (%)
D0	8.9 ± 0.3	15.2 ± 1.0	47.4 ± 2.2	28.5 ± 1.4
D1	9.4 ± 0.1	19.6 ± 0.5	58.1 ± 0.2	13.0 ± 0.3
D2	14.8 ± 5.0	73.5 ± 4.0	not detected	11.7 ± 6.0
PAMAM benchmark sample	not detected	98.9 ± 2.8	not detected	1.1 ± 2.8

a Values are expressed as percentages of the total integrated UV area within the selected 9.5–15.0 min chromatographic interval. Data are reported as mean ± standard deviation from triplicate RP-HPLC-UV analyses performed under identical chromatographic conditions. Individual injection values and integration notes are provided in Table S3.

**Figure 3:**
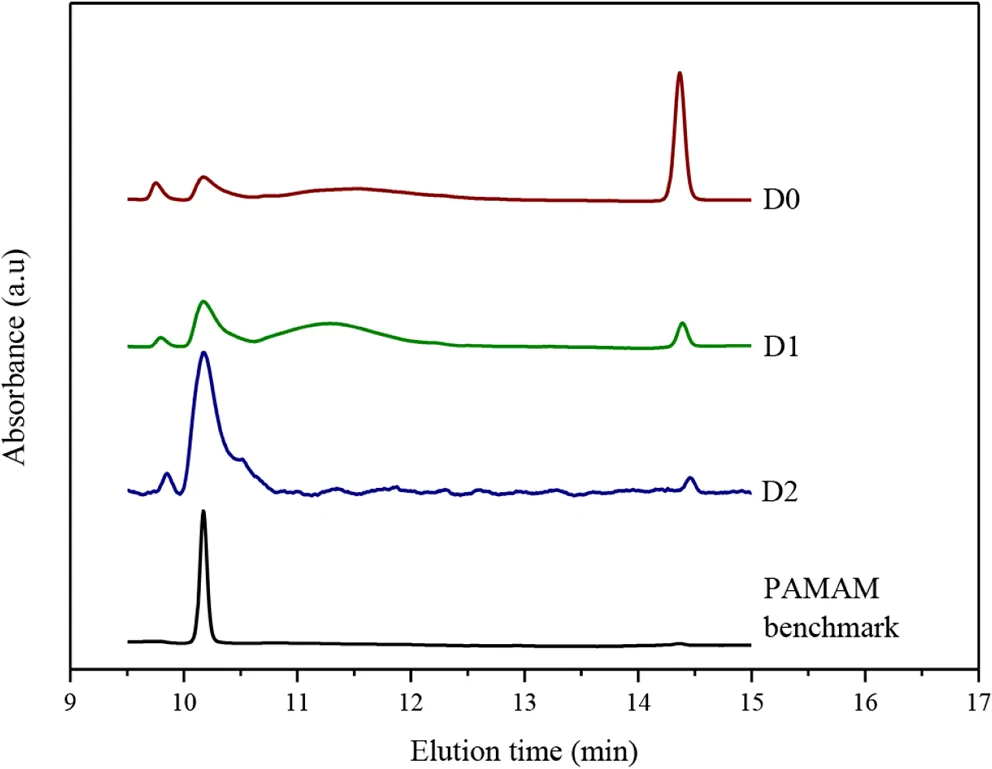
Representative RP-HPLC-UV chromatograms of PAMAM samples synthesized with different EDA excess (D0, D1, and D2) and PAMAM benchmark sample.

The individual RP-HPLC-UV chromatograms obtained from triplicate injections are provided in Figures S4–S7, and the individual retention-window area contributions for each injection are summarized in Table S3.

For D0, the chromatogram exhibited a low relative UV area contribution from the main 10.0–10.5 min retention window, accounting for 15.2 ± 1.0% of the integrated UV area, and a pronounced late-eluting contribution at 14.0–14.5 min, accounting for 28.5 ± 1.4%. This is consistent with a highly heterogeneous chromatographic profile containing a substantial relative UV area contribution from strongly retained components, tentatively assigned to oligomeric dendritic byproducts, covalently bridged species, or aggregate-like populations. The relative UV area contribution from the intermediate 10.5–12.8 min region, 47.4 ± 2.2%, further supports incomplete structural uniformity under stoichiometric EDA conditions.

For D1, the relative UV area contribution of the main 10.0–10.5 min retention window increased to 19.6 ± 0.5%, whereas that of the late-eluting 14.0–14.5 min window decreased to 13.0 ± 0.3%. This suggests that moderate EDA excess suppresses the formation of strongly retained oligomeric byproducts. However, the large relative UV area contribution from the 10.5–12.8 min region, 58.1 ± 0.2%, suggests that D1 still shows a substantial chromatographic response associated with intermediate defect-related species, possibly including incompletely converted, partially branched, or structurally rearranged PAMAM derivatives.

For D2, the chromatographic profile was dominated by the 10.0–10.5 min retention window, which accounted for 73.5 ± 4.0% of the integrated UV area under the selected chromatographic conditions. The intermediate 10.5–12.8 min region was not detected, and the relative UV area contribution of the late-eluting 14.0–14.5 min window decreased to 11.7 ± 6.0%. This profile closely approached that of the PAMAM benchmark sample, which showed 98.9 ± 2.8% of the integrated UV area in the main 10.0–10.5 min window and only 1.1 ± 2.8% in the late-eluting window. These results support the conclusion that the use of high EDA excess substantially improves chromatographic homogeneity and reduces the relative UV area contribution of retention windows assigned to defect-related or strongly retained components.

Importantly, the PAMAM benchmark sample was included only as an independent chromatographic comparison material to evaluate retention profile and relative homogeneity. It was not used to assign absolute purity, calibrate response factors, determine molecular weight, quantify molar fractions, or establish absolute species abundance. The triplicate chromatograms of the PAMAM benchmark sample are shown in Figure S7, and the corresponding relative UV area contributions are included in Table S3.

Complementary SEC analysis revealed that samples with higher EDA excess exhibited SEC profiles consistent with lower apparent hydrodynamic size and narrower apparent size distributions, as shown in Figure 4. SEC data were interpreted cautiously because PAMAM dendrimers possess compact, globular architectures that differ substantially from the linear polymer standards used for calibration. D0 and D1 samples displayed broadly comparable retention-volume profiles, with D0 showing a slightly broader or more heterogeneous profile, although the distinction relative to D1 was modest. D1 showed a similar overall profile to D0, suggesting that moderate EDA excess did not strongly alter the apparent size distribution under the conditions analyzed. In contrast, D2 exhibited the narrowest overall SEC profile among the samples, supporting the trend that high EDA excess reduces apparent size heterogeneity. However, a weak residual contribution or shoulder in the high-apparent-size/early-eluting region cannot be fully excluded for D2. Therefore, the SEC results indicate a reduction, rather than complete elimination, of higher-apparent-size populations when large EDA excess is used.

**Figure 4:**
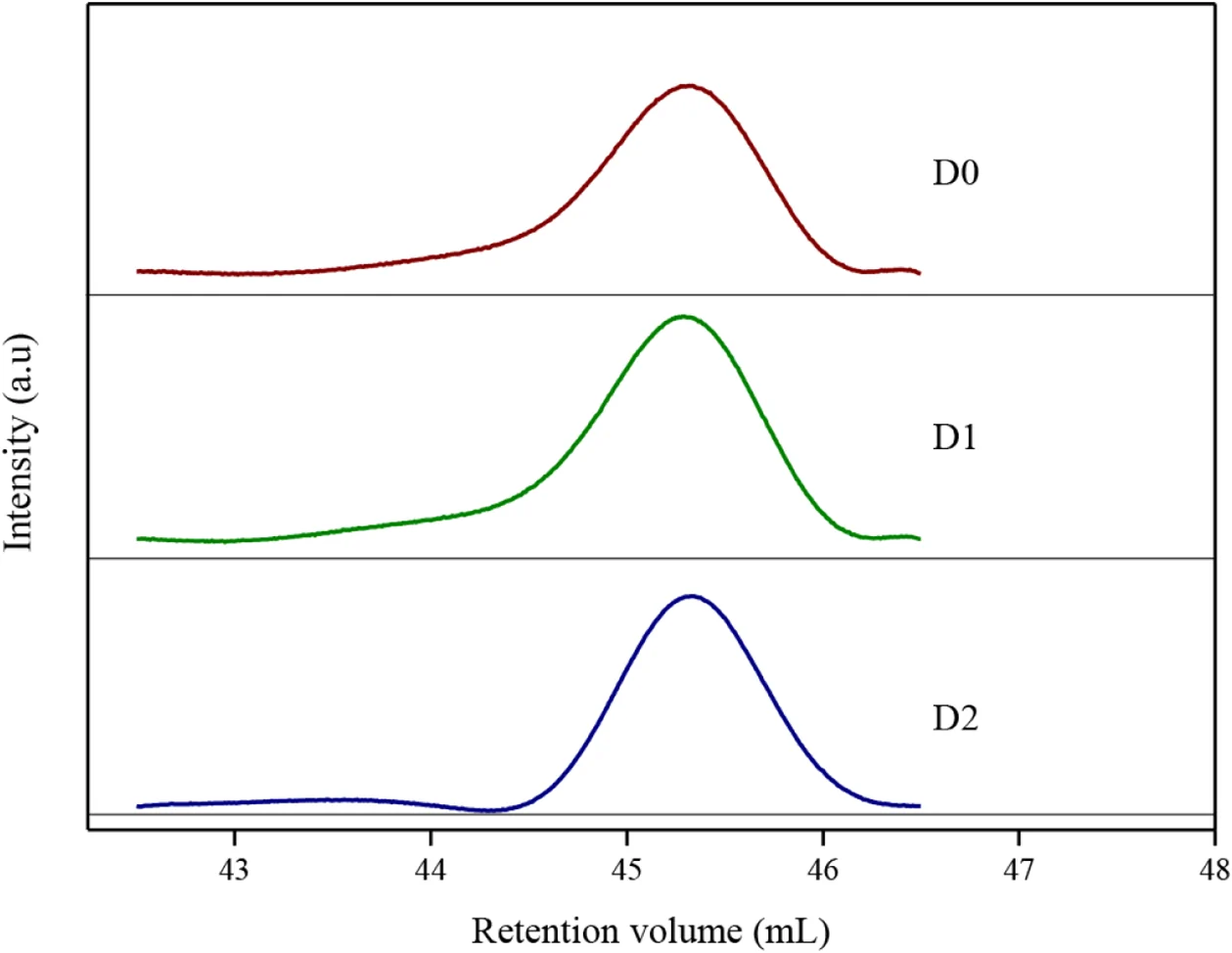
SEC chromatograms of PAMAM synthesis: D0, D1 and D2.

Although all SEC analyses were performed at the same nominal sample concentration, injection volume, and approximate column loading, a concentration-dependence study was not performed. In addition, SEC peak width may be affected by analyte–column interactions and architecture-dependent deviations from ideal size-exclusion behavior. Therefore, SEC peak width was not used as independent quantitative evidence of dispersity or species abundance, nor were SEC profiles used to assign specific molecular species. Instead, SEC was used only as a qualitative descriptor of relative apparent hydrodynamic-size heterogeneity under identical chromatographic conditions, interpreted together with MALDI-TOF MS, RP-HPLC-UV, and ^1^H NMR data.

The SEC-derived molecular-weight distributions are provided in Figure S8.

The ^1^H NMR spectra provided characteristic structural assignments for PAMAM dendrimers and defect-related dendritic species, as shown in Figure 5. Distinct resonances were observed at 2.40 ppm (H-3, aliphatic methylene protons adjacent to amide carbonyl), 2.57 ppm (H-1, EDA core methylene protons), 2.69 ppm (H-6, terminal methylene protons near primary amines), 2.77 ppm (H-2, methylene protons adjacent to tertiary amines), 3.21 ppm (H-5, methylene protons adjacent to amide groups in branched, non-cross-linked environments), and 3.27 ppm (H-7, methylene protons within cross-linked amide structures). The corresponding ^1^H NMR integration values used for this estimation are provided in Table S4.

**Figure 5:**
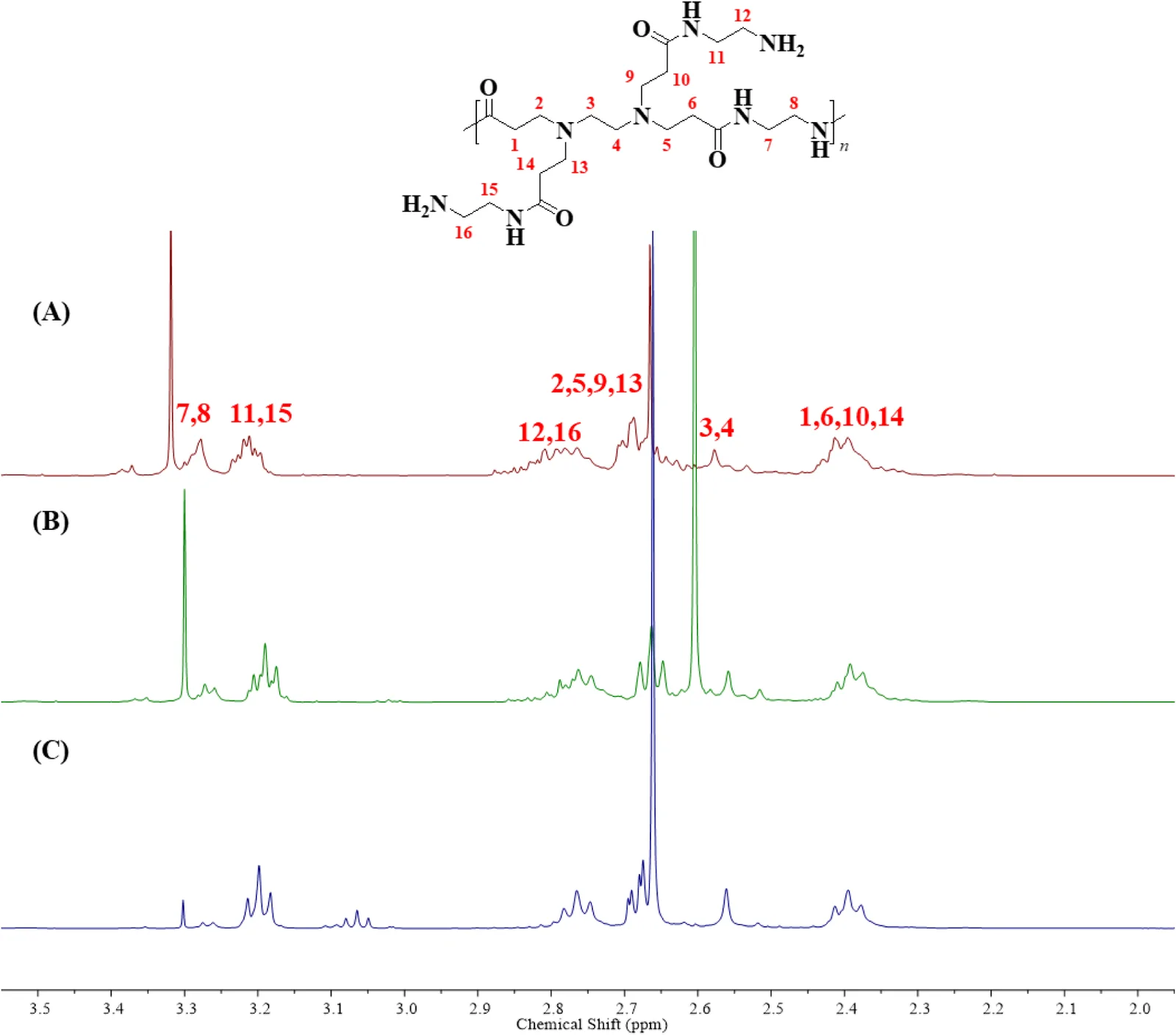
Overlay of ^1^H NMR spectra for PAMAM dendrimers and defect-related dendritic species. (A) D0, (B) D1, and (C) D2.

Increased signal intensities at 2.57 and 3.21 ppm, along with a decrease at 3.27 ppm, are consistent with reduced defect-related NMR contributions and a higher apparent contribution from non-bridged dendritic environments. Specifically, the increase in the 2.57 ppm signal is consistent with a higher relative contribution from EDA-derived methylene environments associated with the dendrimer core or terminal amine-containing segments, whereas the 3.21 ppm resonance is assigned to methylene groups adjacent to amide groups in branched, non-cross-linked environments. Conversely, the 3.27 ppm signal is associated with methylene environments affected by bridged or cross-linked amide structures. Therefore, the increase of the 2.57 and 3.21 ppm resonances combined with the decrease of the 3.27 ppm resonance is consistent with improved branching efficiency and reduced covalent bridging.

Integration of the methylene signals associated with amide groups enabled estimation of the *f*_0_, which was compared to theoretical estimates obtained from a statistical expression adapted from Tomalia.^1^ The *f*_0_ values derived from NMR integration followed the same increasing trend as the theoretical estimates, with a descriptive R^2^ = 0.9588 based on three samples only, suggesting that this statistical treatment may be used as a preliminary descriptor of the observed stoichiometric trend in branching-related environments and defect-related signatures. The semi-quantitative NMR-derived and theoretical *f*_0_ estimates are summarized in Table S5.

Linear regression analysis yielded the equation *f*_o,exp_ = 1.0242· *f*_o,theo_–0.1114, with a mean absolute percentage error (MAPE) of 13.67%. These deviations may arise from limitations in signal resolution during integration, sample impurities, or structural imperfections not accounted for by the model. Nevertheless, the observed agreement is consistent with the expected stoichiometric trend and highlights the utility of ^1^H NMR for semi-quantitatively estimating branching efficiency in dendritic architectures.

Because this correlation was calculated from only three experimental samples, D0, D1, and D2, it should not be interpreted as statistical validation. Rather, the agreement between experimental and theoretical values indicates a useful preliminary trend that is consistent with the expected effect of EDA stoichiometry. A larger dataset containing additional EDA ratios would be required to test this relationship quantitatively.

The statistical treatment assumes comparable reaction rates for amidation leading to dendrimer growth and bridge formation, allowing a simplified theoretical estimate of the expected trend based solely on the molar ratio of reagents. Even under conditions of 650% molar excess of EDA (D2), the theoretical estimate and NMR-derived descriptors indicated a residual apparent bridged contribution of approximately 0.1, suggesting that complete suppression of bridging was not observed under the conditions investigated. This limitation may arise from steric hindrance, conformational constraints, or differential reactivity among terminal groups.

Moreover, this simplified statistical treatment does not account for molecular defects such as intramolecular cyclization or premature terminations, which can compete with dendrimer and linked-species growth, particularly in divergent synthesis strategies. While excess EDA statistically reduces bridge formation and minimizes cyclization, it does not eliminate these side reactions entirely.

Overall, the observed agreement between experimental and theoretical *f*_0_ values highlights the pivotal role of reagent stoichiometry in dictating the branching architecture of PAMAM-based materials. Under stoichiometric conditions or low EDA excess, intermolecular bridging, incomplete branching, and structural heterogeneity are favored, leading to increased formation of covalently bridged species and oligomeric dendritic byproducts. In contrast, higher EDA excess is associated with analytical signatures consistent with improved ester-group conversion, reduced bridging- and cyclization-related responses, and a higher apparent contribution from chromatographically and spectroscopically more homogeneous dendritic products.

From a practical standpoint, the use of large EDA excess is an effective but not trivial synthetic strategy. Although 650% EDA excess improved the structural profile of D2, this approach increases reagent consumption, cost, solvent demand, and purification burden. Although reduced-pressure treatment was applied after the amidation step, residual EDA and methanol were still detected by ^1^H NMR, indicating that this procedure was insufficient for complete removal of low-molecular-weight components under the conditions employed. In practical syntheses, more effective removal of residual EDA may require additional procedures, such as repeated co-evaporation with volatile solvents, azeotropic distillation, prolonged high-vacuum drying, dialysis, or ultrafiltration, depending on the molar mass and solubility of the target product. However, for low-generation PAMAM-related products, purification must be carefully optimized, as their low molar mass and high hydrophilicity can lead to product loss, particularly in dialysis- or ultrafiltration-based protocols. Residual EDA can be monitored by ^1^H NMR through its characteristic methylene resonances. Therefore, the synthetic benefit of excess EDA must be balanced against purification efficiency, process scalability, environmental impact, and final product specifications.

These findings emphasize the importance of precise stoichiometric control to increase dendrimer-related analytical responses and reduce defect-related signatures during divergent synthesis.

### 3.2 Molecular Weight and Dispersity

In this section, the terms molecular weight and dispersity are used only in the sense of apparent, technique-dependent descriptors. Apparent molecular-weight-related descriptors obtained by SEC, MALDI-TOF MS, and ^1^H NMR, were compared with Carothers-type theoretical trends for the synthesized PAMAM dendrimer-related products and defect-related dendritic populations. RP-HPLC-UV was not used to determine molecular weight. Instead, it was used to compare chromatographic fingerprints and relative sample heterogeneity.

The comparison between Carothers-type theoretical estimates and experimentally derived descriptors is summarized in Table 2. The Carothers-type estimates of apparent *M*_*n*_ and *M*_*w*_ are summarized in Table S6. The expected ^1^H NMR integration patterns for apparent series indices are provided in Table S7, and the interpolation used to obtain the apparent NMR-derived molecular-weight descriptor is shown in Table S8. These values were not treated as absolute molecular weight distributions, because each technique is affected by method-specific limitations, including dendrimer compactness and architecture-dependent elution behavior in SEC, ionization and mass-dependent response biases in MALDI-TOF MS, and signal overlap in ^1^H NMR. Therefore, the comparison in Table 2 is interpreted as a descriptive, technique-dependent assessment of apparent molecular-weight trends and structural heterogeneity, rather than as an absolute determination of *M*_*n*_, *M*_*w*_, and *Đ*.

**Table 2:** Experimental and Theoretical Molecular Weight and Dispersity Data for PAMAM Dendrimers and Defect-Related Dendritic Populations^a^

Sample Code	*M*_*n,*theo_ (g mol^–1^)^b^	*M*_*w,theo*_ (g mol^–1^)^b^	*Đ*_theo_ ^b^	*M*_*n,*MALDI_ (g mol^–1^)^c^	*M*_*w,*MALDI_ (g mol^–1^)^c^	*Đ*_MALDI_ ^c^	*M*_*n,*SEC_ (g mol^–1^)^d^	*M*_*w,*SEC_ (g mol^**–1**^)^d^	*Đ*_SEC_ ^d^	*M*_*n,*NMR_ (g mol^–1^)[Table-fn tbl2fn5]
D0	1412.93	2333.53	1.65	1148.94	1293.75	1.13	161	178	1.11	2896.06
D1	722.48	952.63	1.32	837.92	896.52	1.07	161	168	1.04	943.22
D2	558.08	623.84	1.12	697.67	745.64	1.07	148	153	1.03	660.69

i Values are apparent, technique-dependent descriptors and should not be interpreted as absolute molecular-weight or dispersity values.

a Calculated using the Carothers equation.

b Determined by MALDI-TOF MS, considering the *m/z* range defined by the first and last peaks with a relative intensity of at least 5%, regardless of the intensity of intermediate peaks. MALDI-derived values should be interpreted as semi-quantitative because ion intensities are not directly proportional to molar abundance.

c Determined by SEC in aqueous medium. SEC-derived molecular weights are apparent values based on linear polymer calibration standards and may underestimate compact dendritic species.

d Determined by ^1^H NMR through interpolation based on the ratio of the integrated area of aliphatic methylene protons corresponding to amide groups in the branched fraction to the area of methylene protons corresponding to amide groups in the bridged fraction.

Within this conservative framework, increasing EDA excess was associated with lower apparent contributions from higher-mass or defect-related populations and with analytical profiles closer to the expected low-generation PAMAM structure. This trend is consistent with the MALDI-TOF MS, RP-HPLC-UV, SEC, and ^1^H NMR results discussed above, but it should be interpreted as a qualitative to semi-quantitative indication of reduced structural heterogeneity rather than as a rigorous quantitative measurement of dispersity.

The presence of higher-mass or defect-related populations was reflected in the *Đ*, which decreased as the EDA excess increased. Sample D2 exhibited the lowest *Đ* values, indicating a narrow molecular weight distribution and minimal aggregation. In contrast, samples D0 showed broadest distributions and higher *Đ* values, consistent with the presence of structural heterogeneity and linked dendritic byproducts.

SEC measurements yielded significantly lower molecular weight values than expected, likely due to limitations in the calibration curve or reduced sensitivity to low-molar-mass dendrimers. This underestimation, particularly for lower-generation species, is attributed to the compact, globular architecture of dendrimers, which differs from the linear polymer standards used for calibration. SEC also revealed peak broadening and early elution volumes, consistent with, but not definitive proof of, higher-apparent-size populations. These features are consistent with the possible presence of covalently bridged species or oligomeric dendritic byproducts. The retention profile also exhibited a tailing effect, supporting the existence of a heterogeneous population with varying degrees of branching and connectivity, as shown in Figure 4.

In contrast, MALDI-TOF MS provided high-resolution mass distributions, enabling the identification of ideal dendrimer and high-mass bridged species, as well as defect-related dendritic populations. The mass spectra revealed a higher prevalence of structural defects in samples synthesized with lower EDA excess.

^1^H NMR enabled estimation of *M*_*n*_ based on signal integration of methylene protons associated with branching and bridging units, although it does not resolve molecular weight distributions.

Linear regression analyses were conducted to assess the descriptive agreement between Carothers-type theoretical estimates of *M*_*n*_ and *M*_*w*_ and the corresponding apparent molecular-weight descriptors derived from MALDI-TOF MS. For *M*_*n*_, the regression yielded the equation *M*_*n*,exp_ = 0.5051· *Mn*,_theo_ + 429.18, with a coefficient of determination R^2^ = 0.9841 and a MAPE of 17%. For *M*_*w*_, the corresponding equation was *M*_*w*,exp_ = 0.3109· *Mw*,_theo_ + 565.95, with R^2^ = 0.9924 and a MAPE of 23%. Additional details are provided in Figures S1–S3. These results suggest an apparent trend between theoretical estimates and experimental molecular-weight-related descriptors across the three stoichiometric conditions evaluated, as reflected by the high descriptive R^2^ values. However, because the regressions are based on only three data points, these relationships should be interpreted cautiously as preliminary trend descriptors rather than as statistically validated linear correlations. The relatively high MAPE values indicate substantial deviations in magnitude, which may arise from structural imperfections, incomplete dendrimer growth, residual low-molecular-weight components, architecture-dependent analytical bias, simplifications in the Carothers-type model, or limitations inherent to the experimental techniques employed. The presence of constant terms in the regression equations should therefore be interpreted cautiously and may reflect systematic offsets or fitting artifacts rather than physically meaningful constant mass contributions.

Molecular weight distribution calculated using the Carothers equation^32,33^ and compared with SEC and MALDI-TOF MS further support the coexistence of target dendrimer species and defect-related dendritic populations.

Together, these findings demonstrate that reagent stoichiometry plays a critical role in controlling PAMAM architecture. The combined use of mass spectrometry, chromatographic, and spectroscopic data provides an integrated semi-quantitative workflow for comparing structural quality, defect-related signals, chromatographic heterogeneity, and apparent molecular-weight distributions in low-generation PAMAM products.

Rather than claiming absolute quantification or definitive megamer identification, the present workflow highlights the complementary strengths and limitations of each technique. MALDI-TOF MS provides mass-resolved assignments but is affected by ionization bias; SEC compares apparent hydrodynamic-size distributions but is limited by calibration with linear standards; ^1^H NMR estimates branching-related environments but suffers from signal overlap; and HPLC provides chromatographic fingerprints that are useful for comparing heterogeneity and purification efficiency but do not directly yield molecular weights or absolute molar fractions. This combined interpretation supports the conclusion that increasing EDA excess improves the structural quality of low-generation PAMAM dendrimers while reducing defect-related dendritic byproducts.

## 4 Conclusions

This study demonstrates that low-generation PAMAM dendrimer-related products synthesized via a divergent approach are susceptible to structural imperfections, including trailing generations, intramolecular cyclization, and the formation of linked dendritic species, covalently bridged species, and oligomeric dendritic byproducts. The extent of these defects was shown to depend strongly on the stoichiometric ratio of EDA to ester terminal groups, with higher EDA excess favoring chromatographically more homogeneous products.

The integration of MALDI-TOF MS, RP-HPLC-UV, SEC, and ^1^H NMR provided complementary semi-quantitative insights into molar-mass-related signatures, chromatographic heterogeneity, apparent hydrodynamic behavior, and branching-related environments. MALDI-TOF MS was useful for assigning target dendrimer-related signals and detecting higher-mass defect-related populations within the accessible *m/z* range, while RP-HPLC-UV provided a complementary semi-quantitative fingerprint of PAMAM-related species, revealing enrichment of the main chromatographic fraction and reduction of late-eluting heterogeneous populations with increasing EDA excess. SEC highlighted the limitations of conventional calibration for compact dendritic architectures. ^1^H NMR provided semi-quantitative estimates of the *f*_0_, which correlated well with theoretical predictions.

Theoretical estimates based on an adapted Carothers-type treatment showed trends consistent with the experimental descriptors, supporting their use as a preliminary comparative framework rather than as a validated predictive model. Overall, the combined MALDI-TOF MS, RP-HPLC-UV, SEC, and ^1^H NMR results indicate that increasing EDA excess reduces defect-related analytical signatures in low-generation PAMAM products. However, because MALDI ion intensities, RP-HPLC-UV peak areas, SEC peak widths, and ^1^H NMR integrations are affected by technique-specific response factors, and because only three stoichiometric conditions were evaluated, these observations should be interpreted as qualitative to semi-quantitative trends rather than as absolute species abundances, absolute molar fractions, or statistically validated predictive models. Therefore, the observed correlations should be regarded as preliminary descriptors of stoichiometry-dependent structural heterogeneity within the experimental scope of D0, D1, and D2, rather than as broadly validated predictive relationships.

These findings underscore the critical role of reagent stoichiometry in directing dendrimer growth and minimizing defect formation. By incorporating operationally assigned linked dendritic species, covalently bridged species, oligomeric dendritic byproducts, higher-mass or strongly retained defect-related populations, and chromatographic heterogeneity patterns into the characterization framework, this work advances the understanding of dendrimer synthesis and provides a foundation for distinguishing between ideal dendritic structures and defect-containing PAMAM-related architectures in future studies.

## Supplementary Material



## Notes

MALDI-TOF MS spectra of D0, D1, and D2; triplicate RP-HPLC-UV chromatograms; SEC-derived apparent molecular-weight distributions; derivation of the operational stoichiometric imbalance parameter *p*; tentative MALDI-TOF MS assignments; triplicate relative UV area contributions; ^1^H NMR integration data; semi-quantitative NMR-derived and theoretical estimates of the apparent fraction of non-bridged species *f*_0_; Carothers-type apparent *M*_*n*_ and *M*_*w*_ estimates; expected ^1^H NMR integration patterns; interpolation of apparent NMR-derived molecular weight descriptors (PDF)

## Abbreviations

PAMAM: poly­(amidoamine)
EDA: ethylenediamine
MA: methyl acrylate
SEC: size exclusion chromatography
HPLC: high-performance liquid chromatography
RP-HPLC-UV: reversed-phase high-performance liquid chromatography with ultraviolet detection
MALDI-TOF MS: matrix-assisted laser desorption ionization time-of-flight mass spectrometry
ESI-MS: electrospray ionization mass spectrometry
NMR: nuclear magnetic resonance
^1^H NMR: nuclear magnetic resonance
MAPE: mean absolute percentage error
*M*_*n*_: number-average molecular weight
*M*_*w*_: weight-average molecular weight
*Đ*: dispersity
