# Supplementary material for: Stoichiometry-Dependent Structural Defects in Low-Generation PAMAM Dendrimers
Source: ACS Omega. 2026 Sep 30;11(40):62026–39. doi: 10.1021/acsomega.6c08154 (PMC13670331; doi:10.1021/acsomega.6c08154)
Supplement: Supplementary file 1 [file ao6c08154_si_001.pdf]

Stoichiometry-Dependent Structural Defects in Low-Generation PAMAM Dendrimers

*Chun T. Huang[1], Cesar L. Petzhold[1] **

[1]Institute of Chemistry, Federal University of Rio Grande do Sul, Av. Bento Gonçalves, 9500, Porto Alegre – RS, 90650-001, Brazil

*petzhold@iq.ufrgs.br

SUPPORTING INFORMATION

The calculations and interpolations presented in Tables S1 and S5–S8 are intended to provide transparent semi-quantitative descriptors for comparing stoichiometric trends among D0, D1, and D2. The parameters p, f_o, M_n and M_w should not be interpreted as independently validated physical quantities or absolute molecular-weight/species-abundance values. Instead, they are used as operational, theoretical, or apparent descriptors to support comparison with MALDI-TOF MS, RP-HPLC-UV, SEC, and ^{1}H NMR trends.

Table S1. Derivation of the operational stoichiometric imbalance parameter p used for Carothers-type molecular-weight estimates.

Sample Code	n_{EDA}	n_{ester}	$n_{amine}=2n_{EDA}$	Calculation of p	p
D0	4	4	8	$\frac{4+4}{4+8}$	0.667
D1	10	4	20	$\frac{4+4}{4+20}$	0.333
D2	30	4	60	$\frac{4+4}{4+60}$	0.125

The parameter p is derived from the initial functional-group stoichiometry and should not be interpreted as an experimentally measured conversion or extent of amidation.

Table S2. Tentative MALDI-TOF MS assignments.

Sample Code	Observed *m/z*	Tentative assignment	Mass difference / pattern	Relative ion intensity (%)	Comment
D0	516.280	PAMAM-related monomeric ion	Reference ion	42.9	Assigned as main dendrimer-related signal
D0	684.972	Dimer-derived defect species	(516×2) −(1×60)−(60)−(2 ×114)) pattern	99.5	Tentative assignment
D0	799.180	Dimer-derived defect species	(516×2) −(1×60)−(60)−(1 ×114)) pattern	99.5	Tentative assignment
D0	913.239	Dimer-derived defect species	(516×2) −(1×60)−(60)−(0 ×114)) pattern	65.6	Tentative assignment
D0	973.209	Dimer-related	(516×2) −(1×60)−(0×60)−	20.1	Tentative

		species	(0×114)) pattern		assignment
D0	1429.179	Trimer-related species	(516×3) −(2×60)−(0×60)−(0×114)) pattern	7.7	Tentative assignment
D1	516.246	PAMAM-related monomeric ion	Reference ion	63.4	Assigned as main dendrimer-related signal
D1	684.933	Dimer-derived defect species	(516×2) −(1×60)−(60)−(2×114)) pattern	59.7	Tentative assignment
D1	799.138	Dimer-derived defect species	(516×2) −(1×60)−(60)−(1×114)) pattern	100.0	Tentative assignment
D1	913.149	Dimer-derived defect species	(516×2) −(1×60)−(60)−(0×114)) pattern	40.4	Tentative assignment
D1	973.209	Dimer-related species	(516×2) −(1×60)−(0×60)−(0×114)) pattern	13.8	Tentative assignment
D1	1428.917	Trimer-related species	(516×3) −(2×60)−(0×60)−(0×114)) pattern	1.9	Tentative assignment
D2	516.179	PAMAM-related monomeric ion	Reference ion	100.0	Assigned as main dendrimer-related signal
D2	684.933	Dimer-derived defect species	(516×2) −(1×60)−(60)−(2×114)) pattern	1.8	Tentative assignment
D2	799.054	Dimer-derived defect species	(516×2) −(1×60)−(60)−(1×114)) pattern	7.8	Tentative assignment
D2	913.015	Dimer-derived defect species	(516×2) −(1×60)−(60)−(0×114)) pattern	9.1	Tentative assignment
D2	972.978	Dimer-related species	(516×2) −(1×60)−(0×60)−(0×114)) pattern	19.9	Tentative assignment
D2	1428.003	Trimer-related species	(516×3) −(2×60)−(0×60)−(0×114)) pattern	2.3	Tentative assignment

MALDI-TOF MS assignments are tentative and based on mass-pattern consistency. Relative ion intensities were normalized within each spectrum and should not be interpreted as absolute molar abundances.

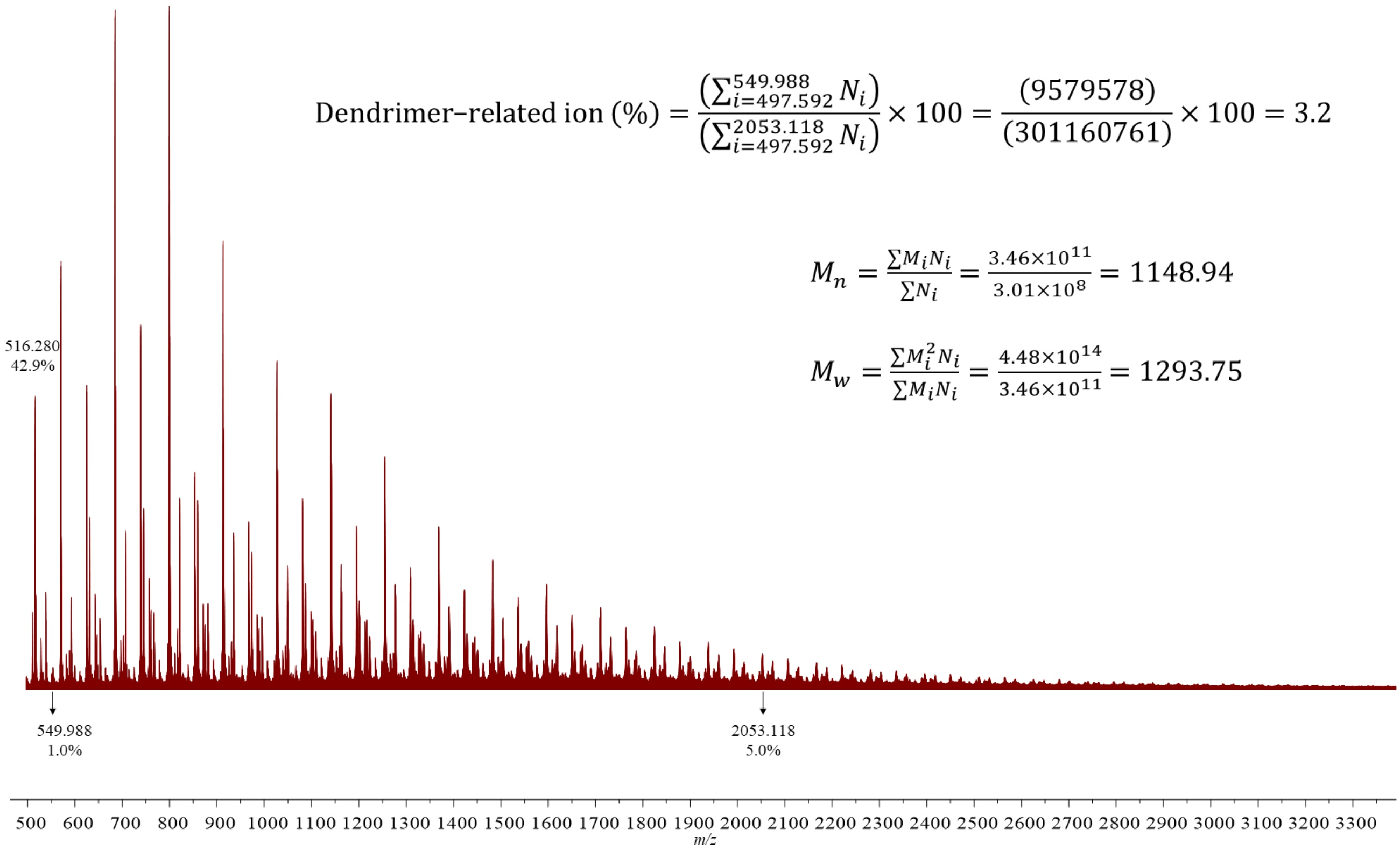

Figure S1. MALDI-TOF mass spectrum of sample D0. The dendrimer-related ion contribution was estimated from the sum of the signal intensities within the *m/z* 497.592–549.988 range, relative to the sum of the total signal intensities within the *m/z* interval defined by the first and last peaks displaying at least 5% relative intensity. Apparent molecular-weight-related descriptors are shown for comparative purposes and should be interpreted as semi-quantitative MALDI-TOF MS descriptors rather than absolute molecular-weight or abundance values.

$$\text{Dendrimer–related ion (\%)} = \frac{\left(\sum_{i=497.592}^{549.988} N_i\right)}{\left(\sum_{i=497.592}^{1369.233} N_i\right)} \times 100 = \frac{(5944023)}{(56546552)} \times 100 = 10.5$$

$$M_n = \frac{\sum M_i N_i}{\sum N_i} = \frac{4.74\times10^{10}}{5.65\times10^{7}} = 837.92$$

$$M_w = \frac{\sum M_i^2 N_i}{\sum M_i N_i} = \frac{4.25\times10^{13}}{4.74\times10^{10}} = 896.52$$

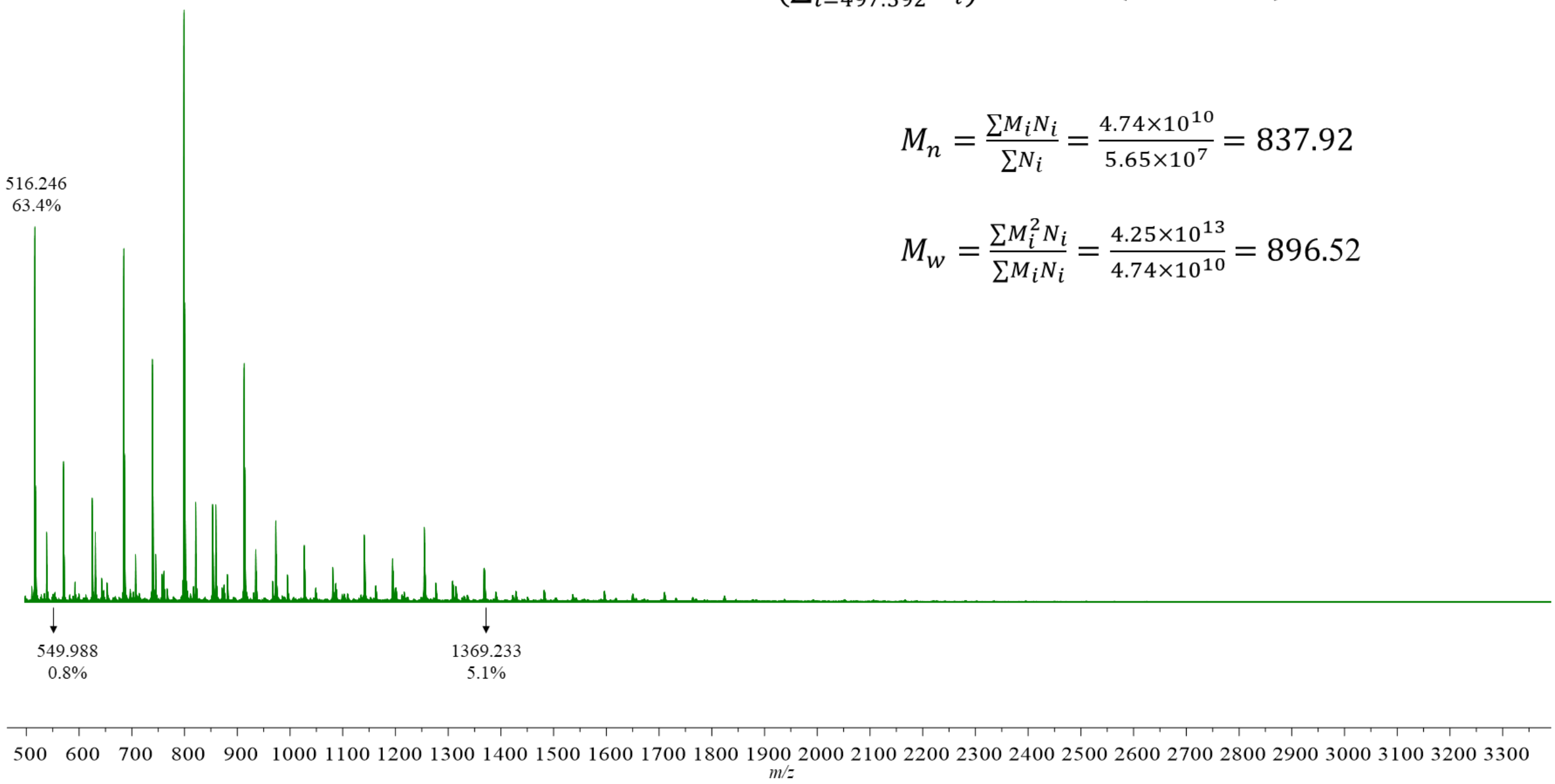

Figure S2. MALDI-TOF mass spectrum of sample D1. The dendrimer-related ion contribution was estimated from the sum of the signal intensities within the *m/z* 497.592–549.988 range, relative to the sum of the total signal intensities within the *m/z* interval defined by the first and last peaks displaying at least 5% relative intensity. Apparent molecular-weight-related descriptors are shown for comparative purposes and should be interpreted as semi-quantitative MALDI-TOF MS descriptors rather than absolute molecular-weight or abundance values.

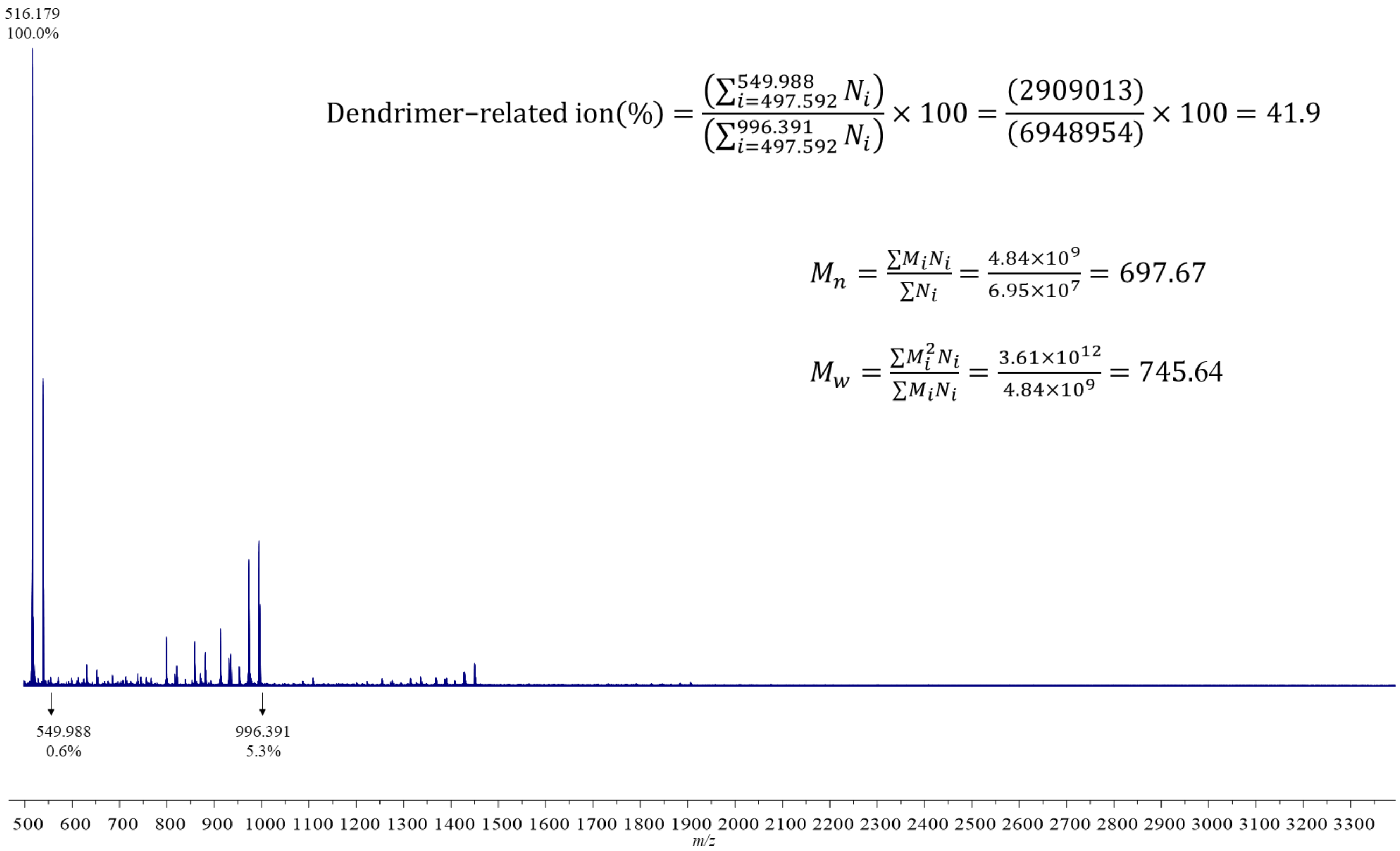

$$\text{Dendrimer-related ion}(\%) = \frac{\left(\sum_{i=497.592}^{549.988} N_i\right)}{\left(\sum_{i=497.592}^{996.391} N_i\right)} \times 100 = \frac{(2909013)}{(6948954)} \times 100 = 41.9$$

$$M_n = \frac{\sum M_i N_i}{\sum N_i} = \frac{4.84\times10^9}{6.95\times10^7} = 697.67$$

$$M_w = \frac{\sum M_i^2 N_i}{\sum M_i N_i} = \frac{3.61\times10^{12}}{4.84\times10^9} = 745.64$$

Figure S3. MALDI-TOF mass spectrum of sample D2. The dendrimer-related ion contribution was estimated from the sum of the signal intensities within the *m/z* 497.592–549.988 range, relative to the sum of the total signal intensities within the *m/z* interval defined by the first and last peaks displaying at least 5% relative intensity. Apparent molecular-weight-related descriptors are shown for comparative purposes and should be interpreted as semi-quantitative MALDI-TOF MS descriptors rather than absolute molecular-weight or abundance values.

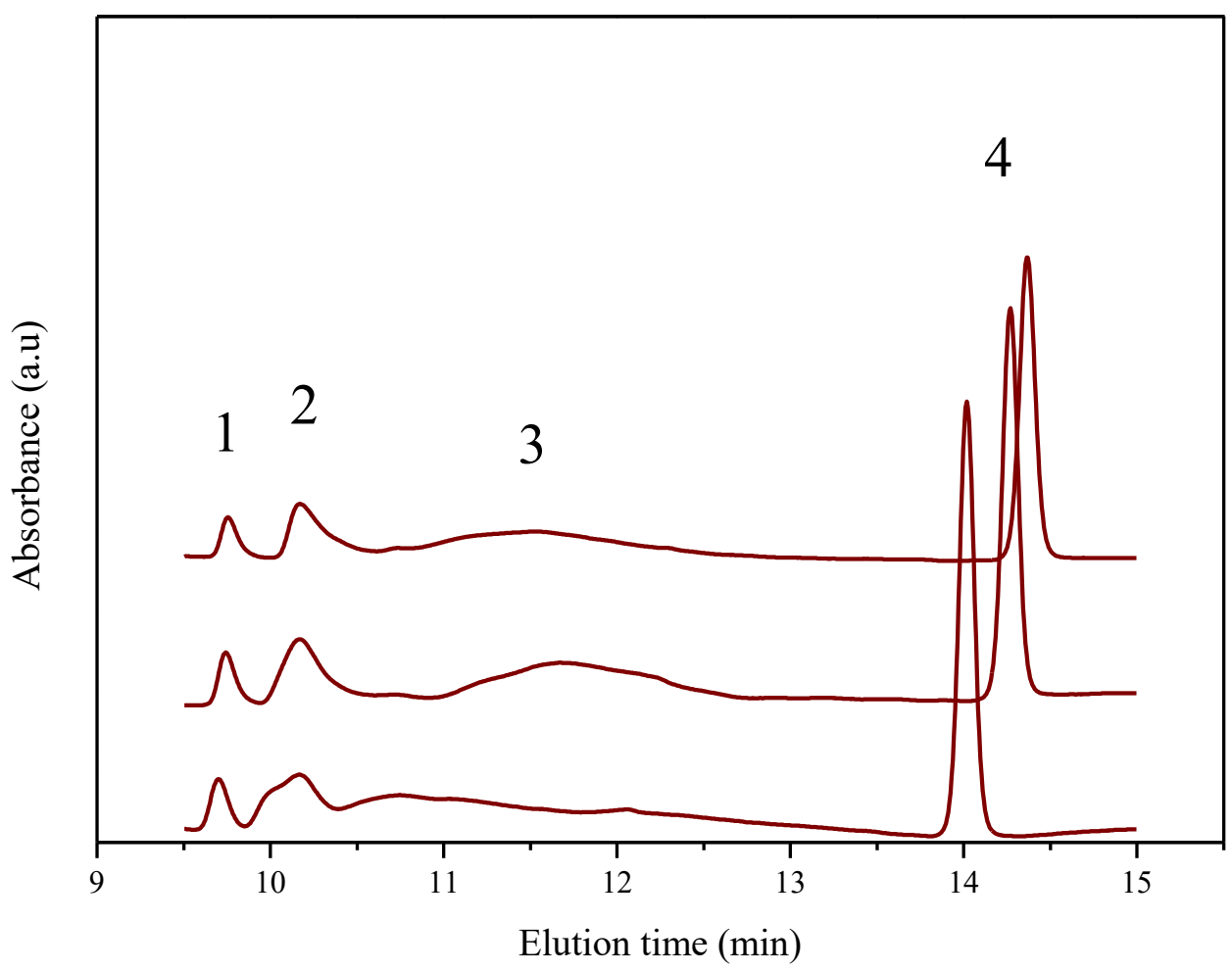

Figure S4. RP-HPLC-UV chromatograms of sample D0 obtained from triplicate injections under identical chromatographic conditions.

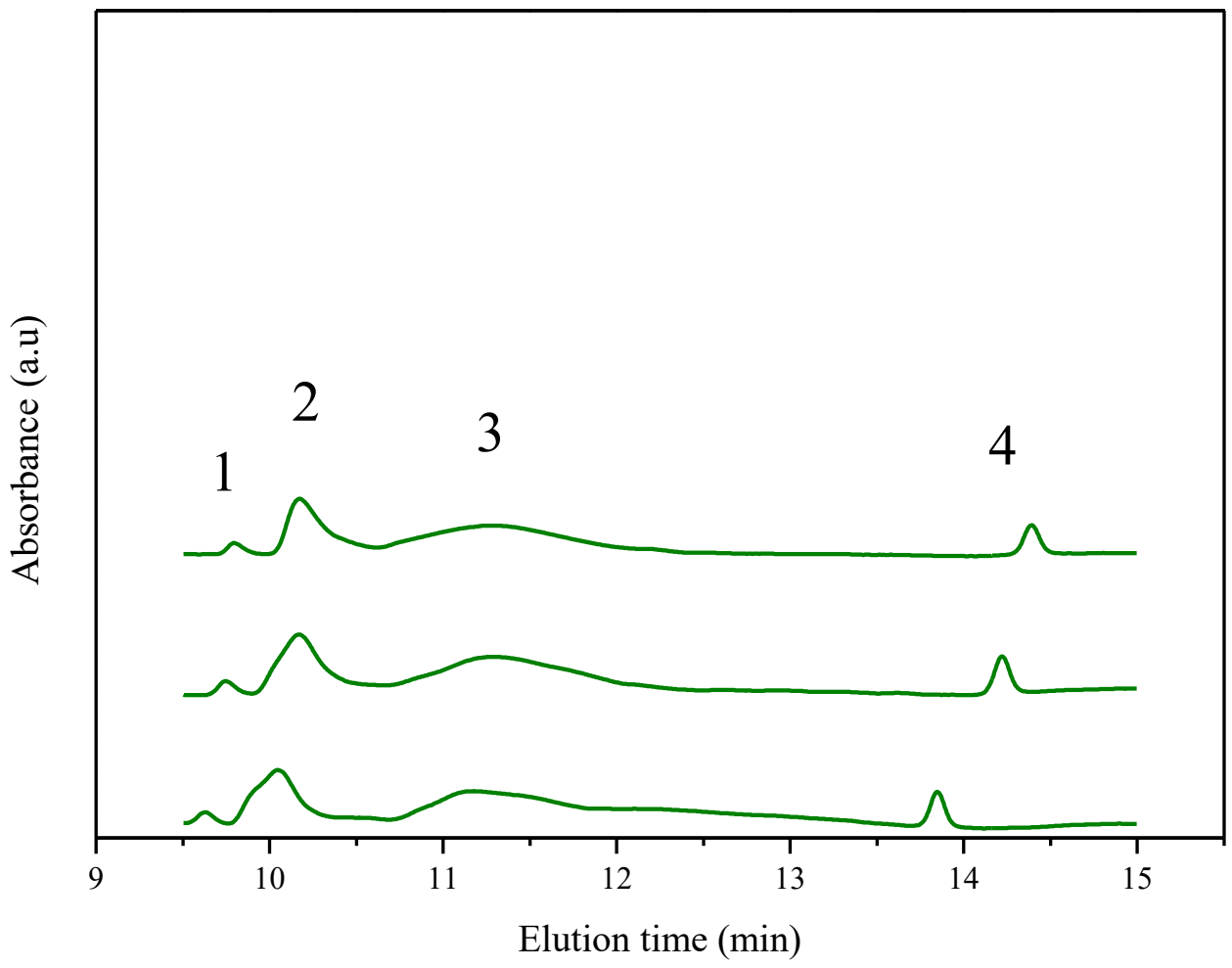

Figure S5. RP-HPLC-UV chromatograms of sample D1 obtained from triplicate injections under identical chromatographic conditions.

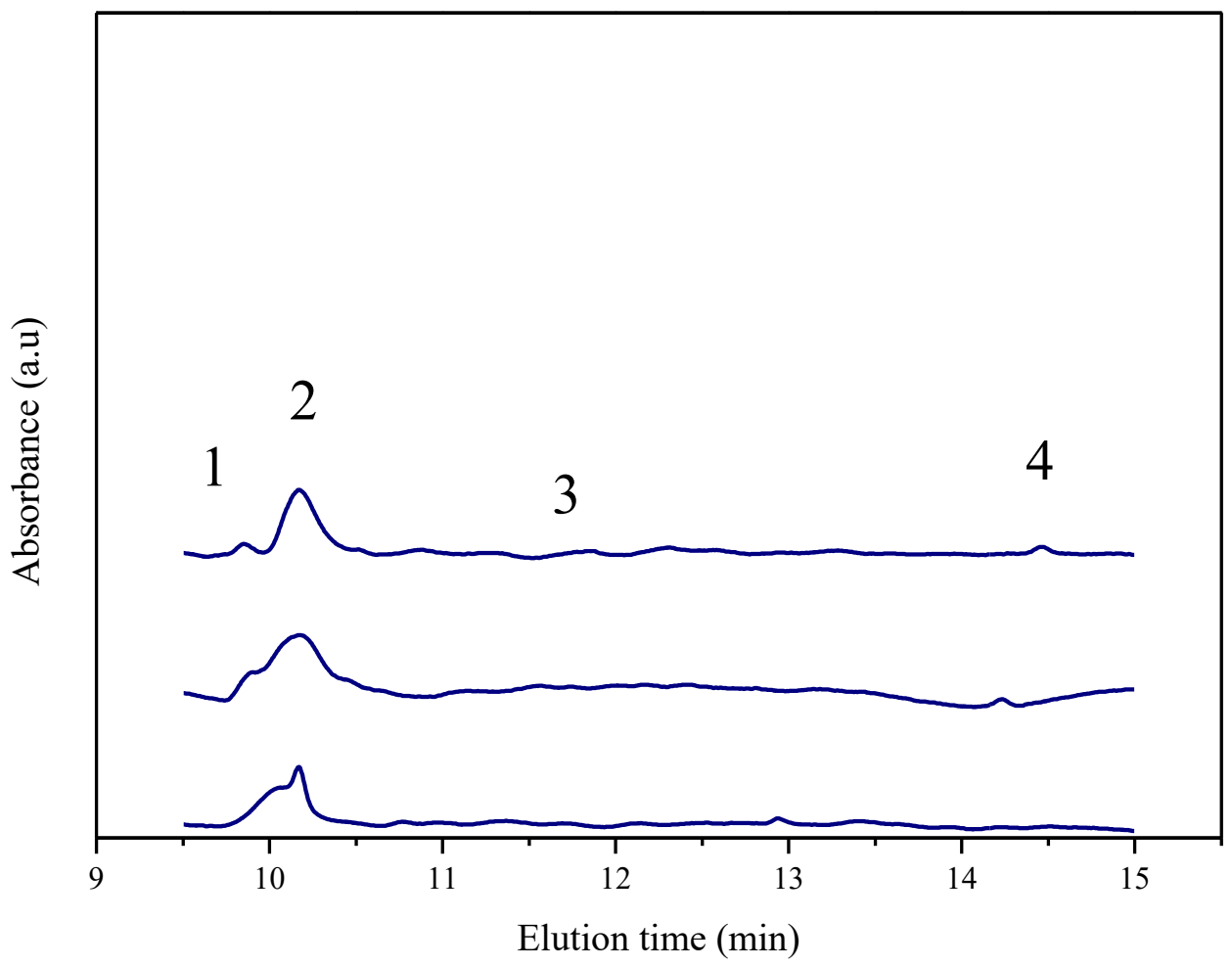

Figure S6. RP-HPLC-UV chromatograms of sample D2 obtained from triplicate injections under identical chromatographic conditions.

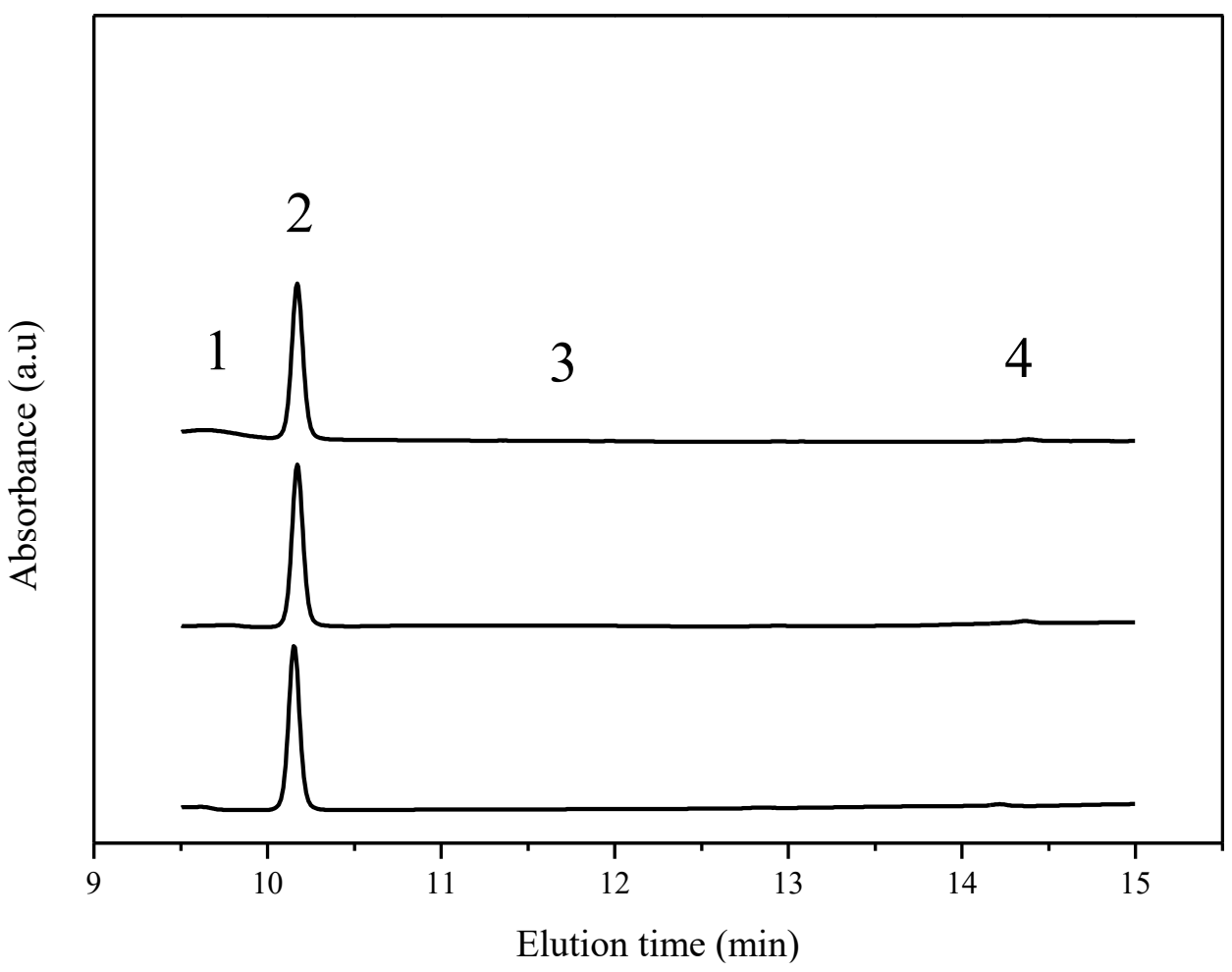

Figure S7. RP-HPLC-UV chromatograms of the PAMAM benchmark sample obtained from triplicate injections under identical chromatographic conditions.

Table S3. Relative UV area contributions obtained from RP-HPLC-UV analyses in defined retention windows.

Sample Code	Peak	Retention time (min)	Relative UV area contribution injection 1 (%)	Relative UV area contribution injection 2 (%)	Relative UV area contribution injection 3 (%)	Mean (%)	SD (%)
D0	1	9.5–10.0	9.0	8.6	9.1	8.9	0.3
D0	2	10.0–10.5	15.7	15.8	14.1	15.2	1.0
D0	3	10.5–12.8	45.9	46.4	50.0	47.4	2.2
D0	4	14.0–14.5	29.4	29.2	26.9[b]	28.5	1.4
D1	1	9.5–10.0	9.3	9.5	9.4	9.4	0.1
D1	2	10.0–10.5	20.1	19.4	19.2	19.6	0.5
D1	3	10.5–12.8	58.0	58.0	58.3	58.1	0.2
D1	4	14.0–14.5	12.6	13.1	13.1[b]	13.0	0.3
D2	1	9.5–10.0	9.3[a]	19.2[a]	15.7[a]	14.8	5.0
D2	2	10.0–10.5	75.7	75.9	68.9	73.5	4.0
D2	3	10.5–12.8	not detected	not detected	not detected	-	-
D2	4	14.0–14.5	15.0	4.9	15.4[b]	11.7	6.0
PAMAM bench mark	1	9.5–10.0	not detected	not detected	not detected	-	-
PAMAM bench mark	2	10.0–10.5	102.7[c]	97.6	96.4	98.9	2.8
PAMAM bench mark	3	10.5–12.8	not detected	not detected	not detected	-	-
PAMAM bench mark	4	14.0–14.5	-2.7[c]	2.4	3.6	1.1	2.8

Relative UV area contributions were calculated independently for each injection and are reported as mean ± standard deviation from triplicate analyses. *a*. The low-retention species was not fully baseline-resolved and was therefore estimated from the shoulder of the main PAMAM-related chromatographic peak. *b*. The late-eluting, strongly retained species shifted partially outside the predefined 14.0–14.5 min retention interval; therefore, its relative area contribution should be regarded as approximate. *c*. Values slightly below 0% or above 100% arise from baseline correction and integration uncertainty in low-intensity chromatographic regions.

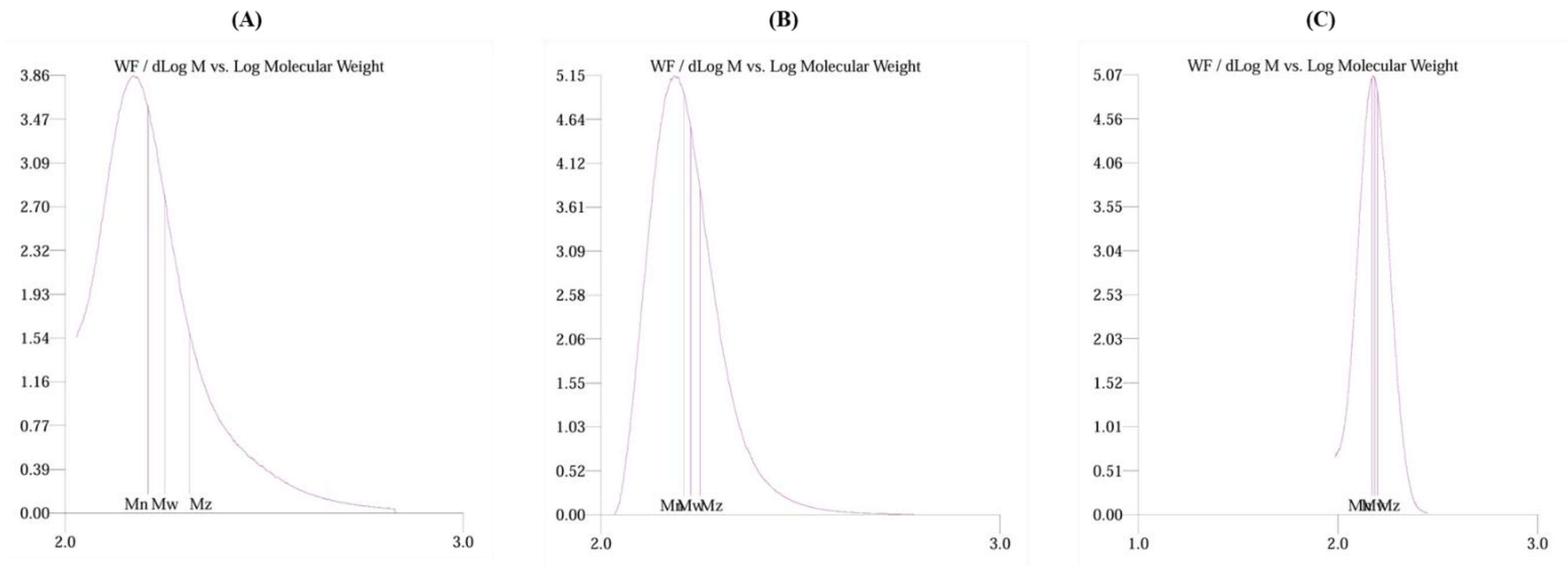

Figure S8. Apparent SEC-derived molecular-weight-related distributions of dendrimer samples. Weight-fraction distributions, expressed as $WF/d\log M$, are plotted as a function of $\log M$. The molecular-weight-related values should be interpreted as apparent descriptors because SEC separation depends on hydrodynamic volume rather than absolute molecular weight. (A) D0, (B) D1, and (C) D2.

Table S4. ^{1}H NMR integration data.

Sample Code	Chemical shift (ppm)	Assignment	Integral area
D0	2.40	Carbonyl-adjacent methylene groups	8.00
D0	2.57	EDA-derived methylene environments	1.69
D0	3.21	Amide-adjacent methylene groups	4.28
D0	3.27	Bridged/cross-linked amide-related environments	3.09
D1	2.40	Carbonyl-adjacent methylene groups	8.00
D1	2.57	EDA-derived methylene environments	2.67
D1	3.21	Amide-adjacent methylene groups	6.19
D1	3.27	Bridged/cross-linked amide-related environments	1.93
D2	2.40	Carbonyl-adjacent methylene groups	8.00
D2	2.57	EDA-derived methylene environments	3.38
D2	3.21	Amide-adjacent methylene groups	7.21
D2	3.27	Bridged/cross-linked amide-related environments	0.76

^{1}H NMR integrations were normalized using the signal corresponding to the carbonyl-adjacent methylene groups.

Table S5. Semi-quantitative NMR-derived and theoretical estimates of the apparent fraction of non-bridged environments, f_o.

Sample Code	Calculation of $f_{o,exp}$[a]	$f_{o,exp}$	Calculation of $f_{o,theo}$[b]	$f_{o,theo}$
D0	(4.28)/(4.28+3.09)	0.5807	$(1-\frac{1}{4\times 1-1})^{4\times 2^{-1}-1}$	0.6667
D1	(6.19)/(6.19+1.93)	0.7623	$(1-\frac{1}{4\times 2.5-1})^{4\times 2^{-1}-1}$	0.8889
D2	(7.21)/(7.21+0.76)	0.9046	$(1-\frac{1}{4\times 7.5-1})^{4\times 2^{-1}-1}$	0.9655

a. Estimated semi-quantitatively from ^{1}H NMR integration as the ratio between amide-adjacent methylene signals assigned to branched/non-bridged environments and the total integrated amide-adjacent methylene signal contribution. Because signal overlap may occur, these values should be interpreted as apparent NMR-derived descriptors rather than absolute molar fractions. *b*. Theoretical values were estimated using a statistical expression adapted from Tomalia *et al.* and are intended to describe the expected stoichiometric trend.

Table S6. Carothers-type estimates of apparent M_n and M_w values based on the operational stoichiometric imbalance parameter p.

Sample Code	p^a	Calculation of $M_{n,\text{theo}}$	$M_{n,\text{theo}}$ (g mol^{-1})	Calculation of $M_{w,theo}$	$M_{w,theo}$ (g mol^{-1})
D0	0.667	$\frac{460.30}{1-0.667}+32$	1412.93	$\frac{460.30\times(1+0.667)}{(1-0.667)}+32$	2333.53
D1	0.333	$\frac{460.30}{1-0.333}+32$	722.48	$\frac{460.30\times(1+0.333)}{(1-0.333)}+32$	952.63
D2	0.125	$\frac{460.30}{1-0.125}+32$	558.08	$\frac{460.30\times(1+0.125)}{(1-0.125)}+32$	623.84

The values of M_n and M_w are theoretical Carothers-type estimates based on the initial stoichiometric imbalance and are not experimentally determined molecular weights. They are used only as comparative descriptors of the expected stoichiometric trend.

Table S7. Expected ^{1}H NMR integration area and molecular weight for apparent series indices.

n^a	δ = 3.27 ppm	δ = 3.21 ppm	δ = 2.57 ppm	δ = 2.40 ppm	$\delta_{3.27}/\delta_{3.21}$	$M_{n,\text{theo}}$ (g mol^{-1})
1	0	8	4	8	0.00	516.39
2	4	12	8	16	0.33	972.70
3	8	16	12	24	0.50	1429.02
4	12	20	16	32	0.60	1885.34
5	16	24	20	40	0.67	2341.65
6	20	28	24	48	0.71	2797.97
7	24	32	28	56	0.75	3254.29

a. Apparent series index used to describe the expected ^{1}H NMR integration pattern.

Table S8. Estimation of an apparent ^{1}H NMR-derived molecular weight descriptor by interpolation of expected integration ratios.

Sample Code	$\delta_{3.27}$/ $\delta_{3.21}$	Calculation of $M_{n,\text{theo}}^{a}$	$M_{n,\text{NMR}}$ (g mol^{-1})
D0	0.72	(2797.97)+((0.72-0.71)×(3254.29-2797.97))/(0.75-0.71)	2896.06
D1	0.31	(516.39)+((0.31-0.00)×(972.70-516.39))/(0.33-0.00)	943.22
D2	0.11	(516.39)+((0.11-0.00)×(972.70-516.39))/(0.33-0.00)	660.69

a. Estimated as an apparent NMR-derived descriptor by interpolation based on the ratio between the integrated area of bridged/cross-linked amide-related methylene protons at 3.27 ppm and branched amide-adjacent methylene protons at 3.21 ppm. These values should not be interpreted as absolute molecular weights determined by ^{1}H NMR.
